# Primary immunodeficiency‐related genes in neonatal intensive care unit patients with various genetic immune abnormalities: a multicentre study in China

**DOI:** 10.1002/cti2.1266

**Published:** 2021-03-22

**Authors:** Tianwen Zhu, Xiaohui Gong, Fei Bei, Li Ma, Jingjing Sun, Jian Wang, Gang Qiu, Jianhua Sun, Yu Sun, Yongjun Zhang

**Affiliations:** ^1^ Department of Neonatology Xinhua Hospital Shanghai Jiao Tong University School of Medicine Shanghai China; ^2^ Department of Neonatology Shanghai Children's Hospital Shanghai Jiao Tong University School of Medicine Shanghai China; ^3^ Department of Neonatology Shanghai Children's Medical Center Shanghai Jiao Tong University School of Medicine Shanghai China; ^4^ Department of Medical Genetics and Molecular Diagnostic Laboratory Shanghai Children's Medical Center Shanghai Jiaotong University School of Medicine Shanghai China; ^5^ Department of Pediatric Endocrinology/Genetics Xinhua Hospital Shanghai Jiao Tong University School of Medicine Shanghai Institute for Pediatric Research Shanghai China

**Keywords:** early life, JAK‐STAT signalling pathway, neonatal intensive care unit, next‐generation sequencing, primary immunodeficiency

## Abstract

**Objectives:**

The present phenotype‐based disease classification causes ambiguity in diagnosing and determining timely, effective treatment options for primary immunodeficiency (PID). In this study, we aimed to examine the characteristics of early‐onset PID and proposed a JAK‐STATopathy subgroup based on their molecular defects.

**Methods:**

We reviewed 72 patients (< 100 days) retrospectively. These patients exhibited various immune‐related phenotypes and received a definitive molecular diagnosis by next‐generation sequencing (NGS)‐based tests. We evaluated the PID‐causing genes and clinical parameters. We assessed the genes that shared the JAK‐STAT signalling pathway. We also examined the potential high risks related to the 180‐day death rate.

**Results:**

We identified PID disorders in 25 patients (34.72%, 25/72). The 180‐day mortality was 26.39% (19/72). Early onset of disease (cut‐off value of 3.5 days of age) was associated with a high 180‐day death rate (*P* = 0.009). Combined immunodeficiency with associated or syndromic features comprised the most common PID class (60.00%, 15/25). Patients who presented life‐threatening infections were most likely to exhibit PID (odds ratio [OR] = 2.864; 95% confidence interval [CI]: 1.047‐7.836). Twelve out of 72 patients shared JAK‐STAT pathway defects. Seven JAK‐STATopathy patients were categorised as PID. They were admitted to NICUs as immunological emergencies. Most of them experienced severe infections and thrombocytopenia, with 4 succumbing to an early death.

**Conclusions:**

This study confirmed that NGS can be utilised as an aetiological diagnostic method of complex immune‐related conditions in early life. Through the classification of PID as pathway‐based subtypes, we see an opportunity to dissect the heterogeneity and to direct targeted therapies.

## Graphical abstract

This study confirmed the utility of next‐generation sequencing (NGS) as a promising approach for the aetiological diagnosis of complex immune‐related conditions in early life. Through the classification of primary immunodeficiency (PID) as pathway‐based subtypes, we see an opportunity to dissect the heterogeneity of PID and to direct the application of targeted therapies.

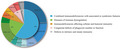

## Introduction

With improvements in health care, genetic diseases have become the leading causes of infant mortality in neonatal intensive care units (NICUs).^1^ Among the life‐threatening genetic conditions in NICUs, immune abnormalities, mostly those predisposing to or causing severe infectious diseases, have frequently been unascertained. Primary immunodeficiency diseases (PIDs)^2^, ^3^, ^4^ are a heterogeneous group of inborn errors of immunity and are mostly caused by monogenic germline mutations. Some of them are uniformly fatal without early definitive therapy within the first few months of life. Advances in next‐generation sequencing (NGS) technologies and clinical care for critically ill infants have resulted in more forms of PID being reported to manifest in early life, the phenotypic spectrum associated with PIDs having evolved and more novel causal genes having been identified, so that infants who would have died in previous eras are now surviving with significant morbidity and mortality.

Currently, the categorisation of PID is based on the clinical, immunological and genetic characteristics of the disease.^5^, ^6^ However, there are often overlapping clinical manifestations of genetic and non‐genetic causes, such as those resulting from compromised immunity,^7^ during early postnatal life, which contribute to the difficulty in defining PID unequivocally and treating it individually. Therefore, the contemporary classification of human disease^8^ was intrinsically limited for this subgroup. With the advent of genomewide molecular diagnostic techniques, identifying the underlying genetic defects has been widely used to classify diseases, predict disease progression and determine effective treatment options.^9^, ^10^, ^11^, ^12^ Some studies^13^, ^14^, ^15^ indicated that mutations in different genes could affect the same cell signalling pathway and result in comparable clinical phenotypes; this finding has helped us understand genomic similarities and the difference between closely related clinical disorders. Accordingly, we hypothesised that paediatric patients who present immune‐related conditions with suspected genetic aetiologies might possess monogenic mutations and might share biological mechanisms involving disease‐associated genes from the same or related pathways. With this, we would connect diseases through their clinical phenotypes and associated genes and guide targeted therapeutic interventions. To date, studies investigating the roles of mammalian/mechanistic target of rapamycin (mTOR)^16^ and nuclear factor‐κB (NF‐κB)^17^ signalling pathways in immune dysregulation diseases have arguably a great effect in the inventories of mechanism‐based therapies, such as infliximab, an anti‐TNF agent in ameliorating autoinflammatory symptoms in patients with A20 haploinsufficiency,^18^ and sirolimus, an mTOR inhibitor used to treat activated phosphoinositide 3‐kinase δ syndrome (APDS).^19^ However, studies on the associations between genetic aberrations in specific pathways and immune deficiencies in critically ill infants are currently sparse, and their clinical implications for precision medicine are building.

In this study, we utilised NGS technology to recognise disease‐causing mutations in PID in a selected cohort of unrelated critically ill infants presenting with various immune‐related conditions in China. We perceived that a life‐threatening infection was the key clinical feature to identify a suspected PID in very early life. We also found that the genetic defects of some patients could be mapped to the Janus kinase (JAK)–signal transducer of activators of transcription (STAT) pathway and that this pathway was shared by some patients with severe PID disorders.

## Results

### Patient characteristics

**Figure 1:**
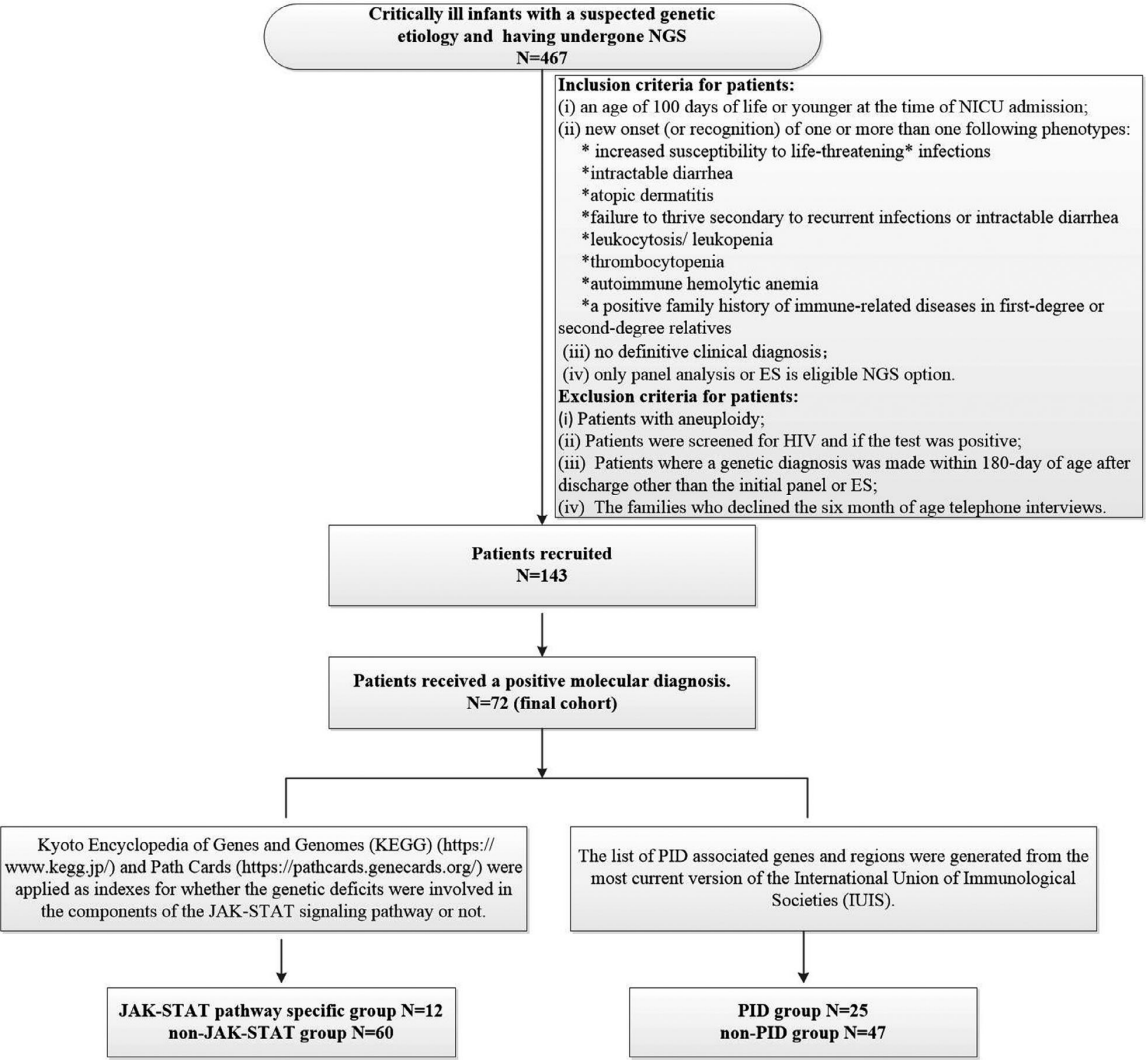
Summary of steps of patient selection and NGS data analysis used in the study. ES, exome sequencing; JAK‐STAT, Janus kinase (JAK)–signal transducer of activators of transcription (STAT); NGS, next‐generation sequencing; NICU, neonatal intensive care unit; PID, primary immunodeficiency. *The patients with life‐threatening infections are eligible if they have sepsis or sepsis shock, or if they need ventilation support or vasopressor therapy or present organ dysfunction, coupled with suspected infection.

Seventy‐two patients fulfilling the designated clinical inclusion criteria were identified as eligible for final analysis (Figure 1). Among these patients, 44 (61.11%, 44/72) were male. NGS method included panel analysis (*n* = 12, 16.67%) and exome sequencing (ES) (*n* = 60, 83.33%). A summary of all pathogenic variant types of 46 genes and nine copy number variations (CNVs) is provided in Supplementary table 1. Consanguinity was denied in 70 cases. Patients were born in nine different provinces in China and were unrelated. The mean ± standard deviation (SD) for birth weight was 3063.06 ± 746.05 g, and the percentage of premature neonates (< 37 weeks of gestation) was 25% (18/72). The clinical phenotypes were present at birth or in early age (0–97 days of age), with a median age of onset at 7.50 days. Although these infants had diverse clinical features, they exhibited one or more of the immune‐related phenotypes at admission or during hospitalisation. In total, 48 patients were vaccinated with BCG and/or hepatitis B within 3 days of birth. No inoculation site infection and left axillary lymphadenectasis co‐occurred with the immune‐related phenotypes. Vaccinations for the remaining 24 patients were delayed because of neonatal onset and admissions to NICUs immediately after birth.

### Genetic spectrum and clinical implications of PID

**Figure 2:**
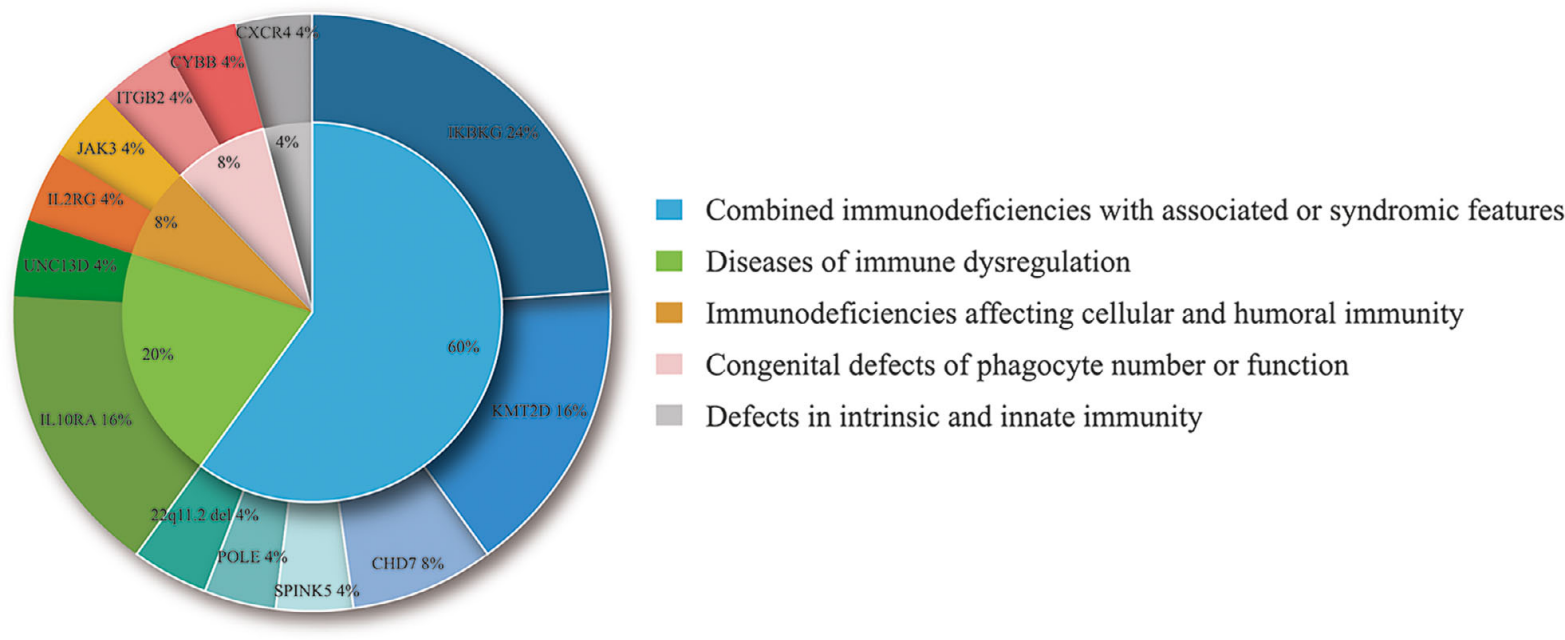
Prevalence of different types of PIDs in the study group (25 patients). PID, primary immunodeficiency.

In total, 25 patients (34.72%, 25/72) were diagnosed with PID based on the molecular aetiology (Table 1). Thirteen different clinical diagnoses were made, which were separated into five groups according to the International Union of Immunological Societies (IUIS) classification.^20^ Figure 2 showed the percentage of patients in each PID group according to the molecular diagnosis. Combined immunodeficiencies with associated or syndromic features were the most common PID (60%, 15/25), followed by diseases of immune dysregulation (20%, 5/25), immunodeficiencies affecting cellular and humoral immunity (8%, 2/25), congenital defects of phagocyte number or function (8%, 2/25) and defects in intrinsic and innate immunity (4%, 1/25).

**Table 1:** Clinical characteristics and variants of 25 PID patients

ID	Gender	Gene	Variants [RefSeq ID]	*In silico* prediction (SIFT/PolyPhen)	Disease [OMIM#]	Inheritance/zygosity	Associated features	IUIS classification	180 days of age outcome
1	M	*JAK3*	c.3050T>C(p.Leu1017Pro) c.1744C>T(p.Arg582Trp) [NM_000215.3]	Deleterious/probably damaging Deleterious/possibly damaging	JAK3 deficiency [OMIM: 600173]	AR/compound het	Life‐threatening infections; atopic dermatitis; thrombocytopenia; anaemia	Immunodeficiencies affecting cellular and humoral immunity/T‐B + SCID	ID at 15 weeks, 6 days
12	F	*IL10RA*	c.301C>T(p.Arg101Trp) c.1283delC(p.Pro428Argfs*20) [NM_001558.4]	Deleterious/probably damaging ‐/‐	IL‐10Ra deficiency [OMIM: 146933]	AR/compound het	Life‐threatening infections; intractable diarrhoea; thrombocytopenia; anaemia	Diseases of immune dysregulation/immune dysregulation with colitis	NND at 26 days
13	M	*IL10RA*	c.301C>T(p.Arg101Trp) c.537G>A(p.=) [NM_001558.4]	Deleterious/probably damaging ‐/‐	IL‐10Ra deficiency [OMIM: 146933]	AR/compound het	Recurrent infections; intractable diarrhoea; atopic dermatitis; failure to thrive secondary to recurrent infections or intractable diarrhoea	Diseases of immune dysregulation/immune dysregulation with colitis	Survival
14	M	*IL10RA*	c.106G>A(p.Ala36Thr) c.299T>G(p.Val100Gly) [NM_001558.4]	Deleterious/probably damaging Deleterious/probably damaging	IL‐10Ra deficiency [OMIM: 146933]	AR/compound het	Recurrent infections; intractable diarrhoea; atopic dermatitis; failure to thrive secondary to recurrent infections or protracted diarrhoea (cytomegalovirus infection)	Diseases of immune dysregulation/immune dysregulation with colitis	Survival
15	M	*IL2RG*	c.421C>T(p.Gln141*) [NM_000206.3]	‐	γc deficiency (common gamma chain SCID, CD132 deficiency) [OMIM: 308380]	XL/inherited hemi	Life‐threatening infections; atopic dermatitis; thrombocytopenia; anaemia	Immunodeficiencies affecting cellular and humoral immunity/T‐B + SCID	ID at 20 weeks
16	M	*CXCR4*	c.685T>A(p.Ser229Thr) [NM_003467.2]	Tolerated/possibly damaging	WHIM (warts, hypogammaglobulinaemia, infections, myelokathexis) syndrome [OMIM: 162643]	AD/inherited het (inherited from mildly affected mother)	Recurrent infections; failure to thrive secondary to recurrent infections; thrombocytopenia; neutropenia; hepatosplenomegaly.	Defects in intrinsic and innate immunity/epidermodysplasia verruciformis (HPV)	Survival
17	M	*ITGB2*	c.817G>A(p.Gly273Arg) [NM_000211.5]	Deleterious/possibly damaging	Leucocyte adhesion deficiency type 1 (LAD1) [OMIM: 600065]	AR/homozygous	Life‐threatening infections; recurrent infections; delayed cord separation; atopic dermatitis; failure to thrive secondary to recurrent infections; anaemia; leucocytosis	Congenital defects of phagocyte number or function/defects of motility	Survival
28	M	22q11.2 del	chr22q11.2 del	‐	Chromosome 22q11.2 deletion syndrome (22q11.2DS) [OMIM: 602054]	AD/het (biological parents unavailable)	Life‐threatening infections (blood culture: *Klebsiella pneumonia*); abnormal faeces; congenital heart defects	Combined immunodeficiencies with associated or syndromic features/thymic defects with additional congenital anomalies	Survival
39	M	*IKBKG*	c.1110delinsTT(p.Ala371Cysfs*24) [NM_003639.4]	‐	EDA‐ID because of IKBKG deficiency [OMIM: 300248]	XL/inherited hemi	Life‐threatening infections; recurrent infections; failure to thrive secondary to recurrent infections; thrombocytopenia. (blood culture: *Klebsiella pneumonia*)	Combined immunodeficiencies with associated or syndromic features/anhidrotic EDA‐ID	ID at 8 weeks, 4 days
41	F	*IKBKG*	Exon 4‐10 del [NM_003639.4]	‐	EDA‐ID because of IKBKG deficiency [OMIM: 300248]	XL/inherited het	Atopic dermatitis	Combined immunodeficiencies with associated or syndromic features/anhidrotic EDA‐ID	Survival
46	F	*SPINK5*	c.377_378del(p.Tyr126*) c.2468dup(p.Lys824Glufs*4) [NM_006846.4]	‐ ‐	Comel‐Netherton syndrome [OMIM: 605010]	AR/compound het	Life‐threatening infections; atopic dermatitis; anaemia	Combined immunodeficiencies with associated or syndromic features/hyper‐IgE syndromes (HIES)	ID at 5 weeks, 5 days
47	F	*IKBKG*	Exon 4‐10 del [NM_003639.4]	‐	EDA‐ID because of IKBKG deficiency [OMIM: 300248]	XL/Inherited het	Atopic dermatitis	Combined immunodeficiencies with associated or syndromic features/anhidrotic EDA‐ID	Survival
48	M	*CHD7*	c.6292C>T(p.Arg2098*) [NM_017780.4]	‐	CHARGE syndrome because of CHD7 deficiency [OMIM: 608892]	AD/de novo het	Recurrent infections; anaemia; heart anomaly; choanal atresia; genital and ear anomalies	Combined immunodeficiencies with associated or syndromic features/thymic defects with additional congenital anomalies	Survival
52	M	*KMT2D*	c.14382G>A(p.=) [NM_003482.3]	‐	Kabuki syndrome 1 because of KMT2D deficiency [OMIM: 602113]	AD/de novo het	Life‐threatening infections; protracted diarrhoea; abnormal faeces; congenital heart defects	Combined immunodeficiencies with associated or syndromic features/other defects	Survival
61	F	*IL10RA*	c.251C>T(p.Thr84Ile) c.537G>A(p.=) [NM_001558.4]	Deleterious/probably damaging ‐	IL‐10Ra deficiency [OMIM: 146933]	AR/compound het	Life‐threatening infections; recurrent infections; intractable diarrhoea; failure to thrive secondary to recurrent infections or protracted diarrhoea; thrombocytopenia; anaemia	Diseases of immune dysregulation/immune dysregulation with colitis	Survival
63	M	*UNC13D*	c.766C>T(p.Arg256*) c.640C>T(p.Arg214*) [NM_199242]	‐ ‐	UNC13D/Munc13‐4 deficiency (FHL3) [OMIM: 608897]	AR/compound het	Life‐threatening infections; neutropenia; thrombocytopenia	Diseases of immune dysregulation/familial haemophagocytic lymphohistiocytosis (FHL syndromes)	ID at 23 weeks, 6 days
64	M	*IKBKG*	c.662T>C(p.Leu221Pro) [NM_001099856.6]	Deleterious/probably damaging	EDA‐ID because of IKBKG deficiency [OMIM: 300248]	XL/inherited hemi	Life‐threatening infections; recurrent infections; atopic dermatitis; anaemia; (blood culture: *Acinetobacter baumannii*)	Combined immunodeficiencies with associated or syndromic features/anhidrotic EDA‐ID	ID at 19 weeks, 4 days
65	M	*IKBKG*	Exon 4‐10 del [NM_003639.4]	‐	EDA‐ID because of IKBKG deficiency [OMIM: 300248]	XL/het (mosaic mother)	Atopic dermatitis	Combined immunodeficiencies with associated or syndromic features/anhidrotic EDA‐ID	Survival
66	F	*KMT2D*	c.11017del(p.Asn3674Ilefs*75) [NM_003482.3]	‐	Kabuki syndrome 1 because of KMT2D deficiency [OMIM: 602113]	AD/de novo het	Recurrent infections; anaemia; facial abnormalities; congenital heart defects	Combined immunodeficiencies with associated or syndromic features/other defects	Survival
67	F	*IKBKG*	Exon 4‐10 del [NM_003639.4]	‐	EDA‐ID because of IKBKG deficiency [OMIM: 300248]	XL/inherited het	Atopic dermatitis	Combined immunodeficiencies with associated or syndromic features/anhidrotic EDA‐ID	Survival
68	M	*POLE*	c.1181_1182del(p.Gln394Argfs*29) c.3587C>T(p.Thr1196Met) [NM_006231.4]	‐ Tolerated/benign	POLE1 (polymerase ε subunit 1) deficiency (FILS syndrome) [OMIM: 174762]	AR/compound het	Life‐threatening infections; anaemia; facial dysmorphism (cytomegalovirus infection)	Combined immunodeficiencies with associated or syndromic features/DNA repair defects other than those listed in immunodeficiencies affecting cellular and humoral immunity	Survival
69	M	*CYBB*	c.1151 + 1G>A(p.?) [NM_000397.4]	‐	X‐linked chronic granulomatous disease (CGD), gp91phox [OMIM: 300481]	XL/inherited hemi	Life‐threatening infections; atopic dermatitis	Congenital defects of phagocyte number or function/defects of respiratory burst	ID at 24 weeks, 4 days
70	F	*KMT2D*	c.10595T>C(p.Ile3532Thr) [NM_003482.3]	Deleterious/probably damaging	Kabuki syndrome 1 because of KMT2D deficiency [OMIM: 602113]	AD/de novo het	Life‐threatening infections; facial abnormalities; congenital heart defects	Combined immunodeficiencies with associated or syndromic features/other defects	Survival
71	M	*KMT2D*	c.1967del(p.Leu656Argfs*274) [NM_003482.3]	‐	Kabuki syndrome 1 because of KMT2D deficiency [OMIM: 602113]	AD/de novo het	Life‐threatening infections; facial abnormalities; congenital heart defects	Combined immunodeficiencies with associated or syndromic features/other defects	Survival
72	F	*CHD7*	c.6157C>T(p.Arg2053*) [NM_017780.4]	‐	CHARGE syndrome because of CHD7 deficiency [OMIM: 608892]	AD/de novo het	Intractable diarrhoea; heart anomaly; choanal atresia; genital and ear anomalies	Combined immunodeficiencies with associated or syndromic features/thymic defects with additional congenital anomalies	Survival

AD, autosomal dominant inheritance; AR, autosomal recessive inheritance; EDA‐ID, epidermodysplasia immunodeficiency; F, female; hemi, hemizygous; het, heterozygous; hom, homozygous; ID, infant death; IUIS, International Union of Immunological Societies; M, male; NND, neonatal death; SCID, severe combined immune deficiency; XL, X‐linked inheritance.

Overall, NGS revealed 13 different PID disorders associated with 12 genes harbouring 18 disease‐causing variants and 1 CNV (Table 1, Figure 2). Six patients (P41, P39, P47, P64, P65 and P67) were diagnosed with ectodermal dysplasia with immunodeficiency (EDA‐ID) because of *IKBKG* deficiency (OMIM: 300248). Four of them (P41, P47, P65 and P67) presented with skin lesions after birth and were treated with daily applications of emollients; all four patients achieved remission. The remaining two patients (P39 and P64) were initially suspected to have immune deficiency because they manifested severe and recurrent infections. Various antibiotics (ceftazidime, cefepime and meropenem) were applied, but they both died of blood culture (BC)‐positive sepsis during early infancy. P64 was positive for *Acinetobacter baumannii,* and P39 was positive for *Klebsiella pneumonia*. Four patients (P12, P13, P14 and P61) were diagnosed with IL‐10Ra deficiency (OMIM: 146933) caused by various pathogenic variants in the *IL10RA* gene. These patients presented with intractable diarrhoea about 7 days after birth. Mouth ulcers manifested in 2 cases (P14 and P61), and extra‐intestinal manifestations included perianal abscesses (P13, P14 and P61) and perianal skin tags (P14). Four patients with Kabuki syndrome 1 (OMIM: 602113) (P52, P66, P70 and P71) had mutations in the *KMT2D* gene and demonstrated classic facial features and severe or recurrent infections during hospitalisation. Patient P52 presented with severe diarrhoea, pneumonia, cardiac anomalies and heart failure during the neonatal period; he underwent heart surgery at the age of 2 months, and his condition has since improved. NGS confirmed pathogenic variants in *CHD7* in P48 and P72; both patients were diagnosed with CHARGE syndrome (OMIM: 608892) and presented with a combination of cleft palate, heart malformations and immune abnormalities. The remaining nine gene variants, including 1 CNV, were only identified in 1 infant per variant. These gene variants were characterised by various aforementioned immune abnormalities; among them, P17, who was diagnosed with leucocyte adhesion deficiency type 1 (LAD1) (OMIM: 600065), received haematopoietic stem cell transplantation (HSCT) and survived at 180 days of interview. P28 underwent heart surgery and was in clinical remission. Unfortunately, four patients (P1, P15, P59 and P69) with pathogenic variants in *JAK3*, *IL2RG*, *SPINK5* and *CYBB* did not respond to the current treatments; their parents agreed to withdraw them from the treatments, and they passed away soon after.

### Molecular and clinical features of non‐PID patients

A molecular diagnosis of PID could not be made for the remaining 47 patients. Given the aforementioned immune abnormalities, further robust investigation is warranted to assess whether specific immune abnormalities were more likely to be associated with a diagnosis of PID. When we examined the odds ratio (OR) of various immune‐related phenotypes to determine PID (see Supplementary appendix 1 for details) (Supplementary table 2), we found that patients who presented with life‐threatening infection (*P* = 0.049; OR = 2.864; 95% confidence interval [CI]: 1.047‐7.836) were most likely to be diagnosed with PID.

As for the spectrum of the genetic diseases for the non‐PID patients, it included chromosomal anomalies, imprinted diseases and monogenic diseases (Supplementary table 1). Pathogenic variants in *COL17A1* were identified in 5 patients (P7, P8, P9, P10 and P11), who presented with recurrent skin blistering from birth. Three gene variants (*MMACHC*, *ABCC8* and *PTPN11*) and 2 CNVs (11q24.1‐q25 del and Xp11.23‐p11.22 dup) were identified in 2 infants each. The remaining variants or CNVs were only identified in 1 infant per variant. These critically ill infants with different genetic aetiology presented with analogous phenotypes that resembled infections at admission or during hospitalisation.

### Genetic and phenotypic spectra of patients with JAK‐STATopathy

**Figure 3:**
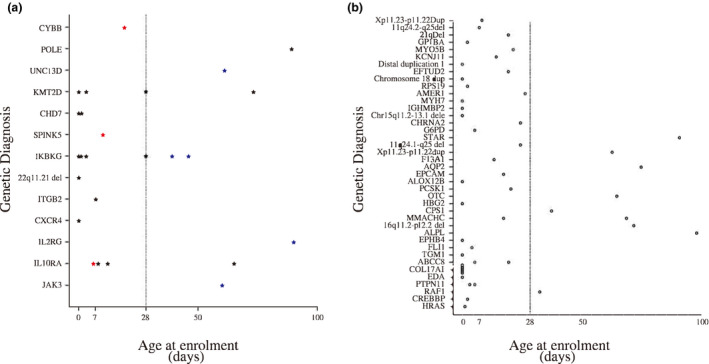
Age at disease onset for 25 PID patients vs 47 non‐PID patients. **(a)** Age at disease onset of 25 PID patients and **(b)** age at disease onset of 47 non‐PID patients. Circles represent non‐PID cases, and stars represent PID cases. Red presents death before 28 DOL, and blue represents death between 28 and 100 DOL. Solid line presents the boundary before 28 DOL and after. DOL, days of life; PID, primary immunodeficiency.

A summary of all pathogenic variants affecting the JAK‐STAT Super Path (indicated by Kyoto Encyclopedia of Genes and Genomes [KEGG] or PathCards) are provided in Table 2, and the identified gene distribution with different ages of onsets is shown in Figure 3a and b.

**Table 2:** The clinical and molecular details in 12 patients with JAK‐STATopathy

ID	Syndrome/disease [OMIM#]	JAK‐STAT pathway gene	Chromosome location	Protein	Protein function	Clinical phenotype	Components of pathway	180 days of age outcome	PID vs non‐PID subgroup
1	JAK3 deficiency [OMIM: 600173]	*JAK3*	19p13.11	JAK3_HUMAN	Tyrosine kinases	Life‐threatening infections; atopic dermatitis; thrombocytopenia; anaemia	1	ID at 15 weeks, 6 days	PID group
2	Costello syndrome [OMIM: 218040]	*HRAS*	11p15.5	C‐Has/Bas P21 protein	GTPase	Recurrent infections; distinctive facial appearance; hypertrophic cardiomyopathy	1	Survival	Non‐PID group
3	Rubinstein‐Taybi syndrome 1 (RSTS1) [OMIM:180849]	*CREBBP*	16p13.3	CREB binding protein	Histone acetyltransferase	Thrombocytopenia seizures	1	Survival	Non‐PID group
4	Noonan syndrome 5 (NS5) [OMIM:611553]	*RAF1*	3p25.2	Raf proto‐oncogene serine/threonine–protein kinase	MAP kinase kinase kinase	Recurrent infections; facial dysmorphic features; congenital heart defects; a short neck.	1	Survival	Non‐PID group
5	Noonan syndrome 1 (NS1) [OMIM:163950]	*PTPN11*	12q24.1	SHP2	Phosphatase	Recurrent infections; intractable diarrhoea; thrombocytopenia; pulmonary valve stenosis; hypertrophic cardiomyopathy	1	ID at 20 weeks, 6 days	Non‐PID group
12	IL‐10Ra deficiency [OMIM: 146933]	*IL10RA*	11q23.3	Interleukin‐10 receptor subunit alpha	Interferon receptors	Life‐threatening infections; intractable diarrhoea; thrombocytopenia; anaemia	2	NND at 26 days	PID group
13	IL‐10Ra deficiency [OMIM: 146933]	*IL10RA*	11q23.3	Interleukin‐10 receptor subunit alpha	Interferon receptors	Recurrent infections; intractable diarrhoea; atopic dermatitis; failure to thrive secondary to recurrent infections or intractable diarrhoea	2	Survival	PID group
14	IL‐10Ra deficiency [OMIM: 146933]	*IL10RA*	11q23.3	Interleukin‐10 receptor subunit alpha	Interferon receptors	Recurrent infections; intractable diarrhoea; atopic dermatitis; failure to thrive secondary to recurrent infections or protracted diarrhoea	2	Survival	PID group
15	γc deficiency (common gamma chain SCID, CD132 deficiency) [OMIM: 308380]	*IL2RG*	Xq13.1	Interleukin‐2 receptor subunit gamma	Interleukin receptors	Life‐threatening infections; atopic dermatitis; thrombocytopenia; anaemia	2	ID at 20 weeks	PID group
16	WHIM syndrome [OMIM: 162643]	*CXCR4*	2q22.1	C‐X‐C chemokine receptor type 4	CXC chemokine receptor	Recurrent infections; failure to thrive secondary to recurrent infections; thrombocytopenia	2	Survival	PID group
61	IL‐10Ra deficiency [OMIM: 146933]	*IL10RA*	11q23.3	Interleukin‐10 receptor subunit alpha	Interferon receptors	Life‐threatening infections; recurrent infections; intractable diarrhoea; failure to thrive secondary to recurrent infections or protracted diarrhoea; thrombocytopenia; anaemia	2	Survival	PID group
62	Noonan syndrome 1 (NS1) [OMIM:163950]	*PTPN11*	12q24.1	SHP2	Phosphatase	Respiratory infections; hypospadias; congenital heart defects	1	Survival	Non‐PID group

Components of JAK‐STAT biological pathway: 1 for cytosol or nucleus of this pathway; 2 for transmembrane receptors of this pathway.

ID, infant death; NND, neonatal death; PID, primary immunodeficiency; SCID, severe combined immune deficiency.

We identified *IL10RA* causal variants in four patients (P12, P13, P14 and P61) (33.33%, 4/12) and *PTPN11* causal variants in two patients (P5 and P62) (16.67%, 2/12). The remaining six variants were identified in 1 infant each (P1, P2, P3, P4, P15 and P16). The affected genes in the 12 patients were categorised as distinguished components of the JAK‐STAT biological pathway based on the corresponding annotation terms. *JAK3, HRAS, CREBBP, RAF1* and *PTPN11* were localised intracellularly of this pathway (JAK‐STAT group 1), while *IL10RA, IL2RG* and *CXCR4* were genes for the corresponding transmembrane receptors of this pathway (JAK‐STAT group 2) (Table 2).

The JAK‐STAT pathway‐associated variants in *JAK3*, *IL10RA*, *IL2RG* and *CXCR4* are loss‐of‐function mutations. In addition, seven patients with these defects can be categorised as PID according to the current criteria. Four cases (P12, P13, P14 and P61) had diseases of immune dysregulation, 2 (P1 and P15) had immunodeficiencies affecting cellular and humoral immunity, and 1 (P16) had defects in intrinsic and innate immunity. We found all of them consistently experienced severe infections (P1, P12, P13, P14, P15, P16 and P61). Four (P12, P13, P14 and P61) were diagnosed with IL‐10Ra deficiency (OMIM: 146933) and treated with various antibiotics; it seemed that supportive treatments such as immunoglobulin transfusion, total parenteral nutrition, concentrated red blood cell transfusion and albumin transfusion to be more effective to treat. A female patient (P12) died at 26 days because of a serious systemic infection, which led to multiple organ failure. The remaining three patients (P13, P14 and P61) were in clinical remission: P13 was treated with thalidomide; P61 was transferred to a gastroenterologist who planned to provide HSCT for him; and P14 was given a highly hydrolysed formula.

Five patients with JAK‐STATopathy (P2, P3, P4, P5 and P62) cannot be categorised as PID. Four patients who were correspondingly diagnosed with Costello syndrome (OMIM: 218040) (P2), Noonan syndrome 5 (OMIM: 611553) (P4) and Noonan syndrome 1 (OMIM: 163950) (P5 and P62) displayed recurrent infections and heart abnormalities constantly. Besides, both P2 and P4 had typical facial features (epicanthal folds, low set and prominent ears). P3 had no signs of infection, but presented with refractory thrombocytopenia and seizures; he possessed a missense variant in the *CREBBP* gene and was diagnosed with Rubinstein‐Taybi syndrome 1 (OMIM: 180849).

Further, we reviewed the routine haematology analysis, immunologic characteristics and microbiological results of these patients. Nine patients (P1, P4, P5, P12, P13, P15, P16, P61 and P62) (43.75%, 9/12) had leucocytosis; 4 (P2, P3, P14 and P16) (33.33%, 4/12) had neutropenia; 6 (P12, P13, P14, P15, P16 and P61) (43.75%, 7/12) had elevated C‐reactive protein (CRP); 2 (P16 and P61) (31.25%, 5/12) suffered from moderate anaemia; and 3 (P3, P5 and P16) (18.75%, 3/12) presented with thrombocytopenia (Supplementary table 3). Laboratory tests revealed leucocyte counts of 1.60–46.37 × 10^9^ L^−1^ (14.93 [9.19–22.63]; reference value: 4–10 × 10^9^ L^−1^), a CRP level < 8 to 160 mg L^−1^ (8.5 [< 8.0–30.5]; reference value < 8 mg L^−1^), a haemoglobin level of 64–177 g L^−1^ (117.94 ± 5.21; reference value: 110–160 g L^−1^) and platelet counts of 15–849 × 10^9^ L^−1^ (321.00 ± 37.70; reference value: 100–300 × 10^9^ L^−1^) (Supplementary table 3). Subgroup analysis revealed no significant difference in leucocyte count, CRP, haemoglobin and platelet count between patients in the two JAK‐STAT pathway‐associated subgroups (JAK‐STAT group 1 vs JAK‐STAT group 2) (data not shown).

Eight patients (P1, P3, P12, P13, P14, P15, P16 and P61) underwent immunologic investigations (Supplementary table 3). A decreased proportion of CD3^+^T cells (reference proportion: 60.8–75.4%) was detected in two patients (P1 and P15). Six patients (P1, P3, P12, P13, P14 and P15) had low levels of serum IgA (reference value: 0.7–4.0 g L^−1^); simultaneously, four of them (P1, P3, P13 and P15) had low levels of IgM (reference value: 0.4–2.3 g L^−1^), and 2 (P1 and P15) had low levels of IgG (reference value: 7–16.0 g L^−1^). P14 had cytomegalovirus (CMV) infection as confirmed by the detection of CMV antibodies and CMV DNA. However, no bacteria were detected in the blood or secretions from these patients after culture.

### The 180‐day outcomes in the final cohort

Overall, 19 patients had died (26.39%, 19/72) before 6 months of age, with four deaths (21.05%, 4/19) occurring in the neonatal period (Supplementary table 1). Ten deceased patients (P5, P12, P20, P25, P45, P46, P49, P54, P56 and P69) (52.63%, 10/19) presented with clinical symptom during the neonatal period (≤ 28 days of life [DOL]). The remaining nine deceased infants (P1, P15, P22, P23, P24, P30, P39, P63 and P64) presented clinically between 28 and 100 DOL (Figure 3a and b). We found that early onset of disease was significantly associated with a high 180‐day death rate (*P* = 0.009) (see Supplementary appendix 1 for details) (Supplementary table 4). The optimal cut‐off value of the age of onset was set at 3.5 days of age (Supplementary figure 1).

Eight patients (P1, P12, P15, P39, P46, P63, P64 and P69) diagnosed with PID died before 6 months of age (Table 1) (Figure 3a). The 180‐day mortality rate was 32.00% (8/25), and all died because of severe infections. They presented with the following symptoms indicating severe infections such as sepsis or sepsis shock or needing ventilation support or vasopressor therapy or presenting organ dysfunction. Two patients (P39 and P64) had positive results of bacterial cultures and no obvious responses to antimicrobial therapies. The statistical analysis revealed no significant association between the mortality and the diagnosis of PID. Moreover, no significant differences were observed when we compared the 180‐day mortality rate with that of non‐PID patients (Supplementary table 4).

Four patients (P1, P5, P12 and P15) (33.33%, 4/12) with genetic deficits in the JAK‐STAT pathway died (Table 2): three died of severe infection without positive results of bacterial cultures and one died of bleeding from a low platelet count. We also found a non‐significant association between the perturbations on this pathway and the 180‐day mortality (Supplementary table 4).

## Discussion

Herein, we report the first multicentre study of a selected cohort of unrelated critically ill infants with various immune‐related conditions in China. Among 72 patients with a definite molecular aetiology, approximately one‐third had PID. Patients who presented with life‐threatening infections were most likely to develop PID. Twelve patients may have shared similar biological mechanisms based on their underlying gene mapping to the same JAK‐STAT pathway. Seven of them were classified with PID and were considered immunological emergencies, with four deceased during hospitalisation or soon after, indicating that this subgroup required targeted therapies rapidly. Finally, we found that the early onset of disease was significantly associated with a high 180‐day mortality.

In this study, we reported 25 newly diagnosed infantile PID patients. Our diagnostic yield (34.72%, 25/72) was similar to that observed in European^21^ and Egyptian patients.^22^ In a recent systematic review, Yska *et al*.^23^ reported that the diagnostic yield of NGS in mixed PID groups ranged from 15% to 46% (median = 25%), which is consistent with that derived from our cohort. When we inspected the associated clinical signs^24^ among patients with PID from NICUs, we found that severe infections were the most common presentations, followed by atopic dermatitis and protracted diarrhoea. As for the immune phenotypes of PID patients, the results of seven patients (P1, P12, P13, P14, P15, P16 and P61) who completed routine immunologic investigations showed limited disease‐associated features (Supplementary table 3). Age‐dependent immunological memory and functional and quantitative deficiencies in antigen‐presenting cells and phagocytes during early life^25^ might explain the high immunological false‐negative results in early‐onset PID. Still, in neonates, the inexperience of antigenic exposure, along with enhancement of tissue‐protective immunosuppressive mechanisms, often results in homogeneous immune‐related symptoms in critically ill patients with different disorders.^25^ This is supported by our findings that 47 non‐PID patients also manifested the aforementioned immune‐related features during early life. Therefore, a timely application of NGS‐based tests in these selected patients is necessary to identify or exclude monogenic PID. NGS‐based tests are beneficial for the application of specific treatments and avoidance of nonspecific and ineffective drugs, thus resulting in an overall reduction in the healthcare costs for this subgroup. Notwithstanding, clinical signs are sometimes nonspecific or masked in the setting of critical conditions in early life. We noted that patients who presented with life‐threatening infections were more likely to be diagnosed with PID; disease‐associated hypogammaglobulinaemia was observed in two severe combined immunodeficiency (SCID) patients (P1 and P15) and one WHIM syndrome patient (P16). Thus, routine haematology and immunologic and microbiological tests may help exclude other causes of the immune‐related conditions in early life and provide some clues for a PID aetiology. In addition, an estimated not‐too‐low potential incidence of SCID in China derived from our study (8.00%, 2/25) and that of Pilania *et al*.^26^ indicates that T‐cell receptor excision circle (TREC) analysis^27^ is an accessible tool for SCID screening. These insights might potentially increase the diagnostic rate of PID and actualise personalised medicine for more patients.

Finally, we explored whether the defects of the JAK‐STAT signalling pathway^28^, ^29^ encompass immune‐related conditions. Our results confirmed that the 12 critically ill infants with genetic defects in JAK‐STAT signalling consistently presented with immune abnormalities. Additionally, seven patients with defects in *IL10RA*, *IL2RG*, *CXCR4* and *JAK3* could be grouped into the PID group. High rates of severe infection (71.43%, 5/7) and thrombocytopenia (71.43%, 5/7) were observed in this group; antibiotics were rendered ineffective. These findings suggest that individuals, who develop a spectrum of similar symptoms, such as recurrent pneumonia, other infections, inflammatory bowel disease and thrombocytopenia, could be the key to determining the underlying genetic defects associated with the JAK‐STAT pathway. The similarity between phenotypes manifesting in different monogenetic diseases in this study reflects the modular nature of related genes that share the same pathway. In addition, the identification of pathway‐specific disease‐causing variants has major therapeutic implications. From our results, SCID (OMIM: 600802), early‐onset inflammatory bowel disease 28 (OMIM: 613148) and X‐linked SCID (OMIM: 312863) should be considered as infantile emergencies. Unfortunately, patients diagnosed with these diseases in our cohort died as a result of severe infections or unsuitable management. However, other studies demonstrated that the survival of children with SCID undergoing cord blood transplantation (UCBT)^30^ or HSCT^31^ before 3.5 months of age HSCT has improved dramatically. Together, these findings reveal that early‐onset PID disorders with aetiologic defects mapped to JAK‐STAT signalling pathway should be considered immunological emergencies necessitating rapid and specific therapies.

The main strengths of this study include the recruitment of biological samples of 72 participants with early‐onset immune‐related conditions and a definitive molecular aetiology, which ensured satisfactory statistical power of the sample and limited the influence of confounding variables that studies with late‐onset patients tend to present. The main limitation is the retrospective design of the study, which is prone to bias. Moreover, NGS diagnostics might fail to detect certain types of variants, such as balanced structure variation and deep intronic variants, which might be a source of minimal distortion. A prospective study with a larger sample size and thorough tests such as routine haematology and immunologic tests, whole‐exome sequencing and/or whole‐genome sequencing in NICU settings would help further validate the utility of NGS as a first‐tier approach in infants with complex immune‐related conditions and provide more in‐depth insight into other sparse signal pathways.

In conclusion, a heterogeneous group of inborn errors of immunity present in infancy and result in significant morbidity and mortality. Therefore, a lower threshold is required for requesting genetic screening when a genetic aetiology is suspected in infants with various immune‐related conditions during the neonatal period or shortly after it. Prompt pathway‐specific categorisation for a group of diseases might be the first step to realise ‘precision medicine’ for NICU patients.

## Methods

### Patient selection algorithm

The patient selection algorithm is summarised in Figure 1. Patients admitted to NICUs at three tertiary hospitals in Shanghai, China: Xinhua Hospital (XH), Children's Hospital (CH) and Shanghai Children's Medical Center (SCMC), between January 2016 and December 2019, were reviewed. All those patients having undergone NGS were collected. Two NGS options were eligible: panel sequencing of the patient who presented specific clinical phenotypes and ES of the patient who presented with multiple congenital abnormalities or syndromic features or genetic conditions of no defined causes. The clinical outcome was the mortality (yes or no) obtained by telephone interview at the age of 6 months. Parents provided written informed consent for the testing itself and agreed to pay for the assay. The analysis and publication of data related to the study were approved by the Medical Ethical Committee of XH Hospital (Approval Number: XHEC‐D‐2020‐164).

Inclusion criteria included the following: (1) less than 100 days, (2) recent presentation with one or more of the following immune‐related conditions: ① increased susceptibility to life‐threatening infections (the patients with life‐threatening infections are eligible if they have sepsis or sepsis shock,^32^, ^33^ or if they need ventilation support or vasopressor therapy or present organ dysfunction, coupled with suspected infection); ② intractable diarrhoea; ③ atopic dermatitis; ④ failure to thrive secondary to recurrent infections or intractable diarrhoea; ⑤ leucocytosis/neutropenia; ⑥ thrombocytopenia; ⑦ autoimmune haemolytic anaemia; ⑧ a positive family history of immune‐related diseases in first‐degree or second‐degree relatives, and (3) no definitive clinical diagnosis. The exclusion criteria were (1) patients with aneuploidy; (2) patients who were positive for HIV screening; (3) patients where a genetic diagnosis was made within 180 days of age after discharge other than the initial panel or ES; and (4) the families who declined the 6‐months‐of‐age telephone interviews.

Each patient was evaluated for enrolment on a case‐by‐case basis after consultation between a clinical geneticist and a neonatologist. Overall, 143 patients were recruited to our data set, where 72 of them were confirmed as having a genetic aetiology. Consequently, 72 patients were included in the final analysis. Data regarding gestational age, birthweight, family history and related clinical and molecular results were extracted from the medical records. The phenotypes of the affected infants were further translated into Human Phenotype Ontology (HPO)^34^ terms (see Supplementary appendix 1 for details).

The list of PID‐associated genes and regions was generated from the most current version of the IUIS.^20^ Seventy‐two subjects were catalysed to PID (PID group *N* = 25) and non‐PID (non‐PID group *N* = 47) subgroups (Supplementary table 1). Concurrently, they were stratified as JAK‐STAT subgroup (JS, *n* = 12) and non‐JAK‐STAT subgroup (NJS, *n* = 60) (Supplementary table 1) based on KEGG (https://www.kegg.jp/) and Path Cards (https://pathcards.genecards.org/).

### Clinical indices

Laboratory evaluations were performed in the study as indicated, including complete blood counts, differential count and CRP. The basic immune components and function were not consistently available owing to the retrospective study design and date of diagnosis in some patients, so that we could not provide a complete immunological analysis for every patient and subgroup comparisons.

### Genetic analysis

DNA was extracted from the peripheral whole blood of 143 patients and their parents, if available, using a QIAamp Blood DNA Mini Kit (Qiagen GmbH, Hilden, Germany). For ES, the capture probes were those used in GenCap Custom Exome Enrichment Kits (MyGenostics, Beijing, China) or TruSight Rapid Capture Kits (Illumina, Inc., San Diego, CA, USA). Thirteen specific disease panels were used (MyGenostics, Beijing, China) (see Supplementary appendix 1 for details). The resulting libraries were sequenced by a HiSeq 6000 platform (Illumina). The Burrows–Wheeler Aligner (BWA) (v.0.5.9‐r16) was used to align the reads to the human reference genome (GRCh37/hg19). CNVs and small variants were identified using VarScan 2 or Genome Analysis Toolkit (GATK) (4.0.10.1).

Potential CNVs identified by WES, if any, were further examined by karyotype testing or chromosomal microarray analysis (CMA). Deleterious variants detected by NGS were confirmed via Sanger sequencing. PolyPhen‐2 and sorting intolerant from tolerant (SIFT) were used to predict the pathogenic effects of unreported variants. Pathogenicity was interpreted according to the five‐tier classification system recommended by the American College of Medical Genetics and Genomics.^35^, ^36^, ^37^

### Statistical analysis

Data were analysed using SPSS 23.0. Categorical variables are displayed as percentiles. The chi‐square analysis was used to compare individual subgroup association with different clinical symptoms. A *P‐*value < 0.05 was considered statistically significant.

## Conflict of interest

The authors declare no conflict of interest.

## Author contributions

**Tianwen Zhu:** Data curation; Formal analysis; Writing‐original draft. **Xiaohui Gong:** Data curation; Writing‐original draft. **Fei Bei:** Data curation; Writing‐original draft. **Li Ma:** Data curation; Writing‐review & editing. **Jingjing Sun:** Data curation; Writing‐review & editing. **Jian Wang:** Data curation; Writing‐review & editing. **Gang Qiu:** Data curation; Writing‐review & editing. **Jianhua Sun:** Data curation; Writing‐review & editing. **Yu Sun:** Conceptualization; Supervision; Writing‐review & editing. **Yongjun Zhang:** Conceptualization.

## Ethical approval

The Institutional Review Boards of Xinhua Hospital, Shanghai Jiao Tong University School of Medicine, approved the study (Approval No. XHEC‐D‐2020‐164).

## Data Availability

The data sets used and/or analysed during the current study are available from the corresponding author Dr. Yongjun Zhang on any reasonable request. All data relevant to the study are included in the article or uploaded as Supplementary Information.
