# Supplementary material for: Primary immunodeficiency‐related genes in neonatal intensive care unit patients with various genetic immune abnormalities: a multicentre study in China
Source: Clin Transl Immunology. 2021 Mar 22;10(3):e1266. doi: 10.1002/cti2.1266 (PMC7984964; doi:10.1002/cti2.1266)

Supporting Information includes:

1. Supplementary Methods: definitions of immune-related conditions in HPO terms; predictors of a positive diagnosis of PID; independent predictors of 180-day mortality.
2. Supplementary Tables: Supplementary table 1, Supplementary table 2, Supplementary table 3, Supplementary table 4.
3. Supplementary Figures: Supplementary figure 1.

(4) The Ethical Review on the Research of “Primary Immunodeficiency Genes in various genetic immunity abnormalities from NICUs: Results of a multicenter study in China”.

**Supplementary Methods**

List of the 3 TES panels (designed by MyGenostics, Beijing, China) for 29 patients in our cohort

| Name for TES Panels | Number of targeted genes involved | Tested Number of patents |
| --- | --- | --- |
| Neonatal metabolism panel | 175 | 16 |
| Digestive system panel | 54 | 7 |
| Immune panel | 232 | 6 |

Abbreviation: TES Targeted Exome Sequencing.

List of all covered genes in the three TES panels

| Name for TES Panels | | | Genes involved in the panel | | | | | | | | | | | | | | | | | | | | | | | | | | | | | | |
| --- | --- | --- | --- | --- | --- | --- | --- | --- | --- | --- | --- | --- | --- | --- | --- | --- | --- | --- | --- | --- | --- | --- | --- | --- | --- | --- | --- | --- | --- | --- | --- | --- | --- |
| Neonatal metabolism panel | | | | |  | | | | | | | | | | | | | | | | | | | | | | | | | | | | |
|  | | | | | *PAH* | | | *L2HGDH* | | *DHTKD1* | | | *CTH* | | | *ARX* | | | *ATP7B* | | | *GCSH* | | | *PTS* | | | *D2HGDH* | | | *GALK1* | | |
|  | | | | | *MTHFR* | | | *SLC6A8* | | *MMAA* | | | *G6PD* | | | *CPS1* | | | *GCH1* | | | *IDH2* | | | *INPP5E* | | | *MTRR* | | | *GAMT* | | |
|  | | | | | *MMAB* | | | *ATP7A* | | *OTC* | | | *QDPR* | | | *ETFA* | | | *LAMP2* | | | *MTR* | | | *GATM* | | | *ABCD4* | | | *PTPN11* | | |
|  | | | | | *ASS1* | | | *PCBD1* | | *ETFB* | | | *MAOA* | | | *GNMT* | | | *ERCC8* | | | *GPHN* | | | *MVK* | | | *ASL* | | | *SPR* | | |
|  | | | | | *ETFDH* | | | *PNPLA2* | | *AHCY* | | | *ERCC6* | | | *MCEE* | | | *ACSF3* | | | *ARG1* | | | *FAH* | | | *BCAT1* | | | *SLC2A1* | | |
|  | | | | | *SLC25A13* | | | *OGDH* | | *OAT* | | | *TAT* | | | *BCAT2* | | | *HSD17B10* | | | *HCFC1* | | | *FH* | | | *NAGS* | | | *HPD* | | |
|  | | | | | *SLC22A5* | | | *SLC2A2* | | *GLUD1* | | | *FOLR1* | | | *LMBRD1* | | | *AASS* | | | *SLC7A7* | | | *HGD* | | | *CPT1A* | | | *SLC3A1* | | |
|  | | | | | *GLUL* | | | *FOLR2* | | *GCDH* | | | *ABHD5* | | | *MAT1A* | | | *HAL* | | | *CPT2* | | | *SLC7A9* | | | *BCKDHA* | | | *DHFR* | | |
|  | | | | | *HMGCL* | | | *ACAT1* | | *CBS* | | | *UROC1* | | | *SLC25A20* | | | *BCKDHB* | | | *DDC* | | | *AUH* | | | *ADK* | | | *SUOX* | | |
|  | | | | | *FTCD* | | | *MLYCD* | | *TYMP* | | | *DBT* | | | *PHGDH* | | | *TAZ* | | | *ALDH6A1* | | | *MOCS1* | | | *GLDC* | | | *ACADSB* | | |
|  | | | | | *TK2* | | | *DLD* | | *PSAT1* | | | *OPA3* | | | *ASPA* | | | *MOCS2* | | | *AMT* | | | *ACADS* | | | *DGUOK* | | | *SARDH* | | |
|  | | | | | *ABAT* | | | *SERAC1* | | *DBH* | | | *NR0B1* | | | *ACADM* | | | *POLG* | | | *PRODH* | | | *ALDH5A1* | | | *FBXL4* | | | *MCCC2* | | |
|  | | | | | *SOX9* | | | *ACADVL* | | *SUCLA2* | | | *ALDH4A1* | | | *SRY* | | | *ALPL* | | | *KMT2D* | | | *PCCA* | | | *CYP21A2* | | | *HADHA* | | |
|  | | | | | *MPV17* | | | *SLC6A20* | | *AR* | | | *PNPO* | | | *KDM6A* | | | *PCCB* | | | *CYP11B1* | | | *HADHB* | | | *C10orf2* | | | *SLC6A19* | | |
|  | | | | | *HSD17B3* | | | *ETHE1* | | *SGSH* | | | *HLCS* | | | *HSD3B2* | | | *HADH* | | | *RRM2B* | | | *SLC36A2* | | | *SRD5A2* | | | *FOXG1* | | |
| Name for TES Panels | | | Genes involved in the panel | | | | | | | | | | | | | | | | | | | | | | | | | | | | | | |
|  | | | *NAGLU* | | | | *BTD* | | | *CYP17A1* | | | *ACAD8* | | *SUCLG1* | | | *IVD* | | | *NR5A1* | | | | *MECP2* | | | *HGSNAT* | | | *PC* | | |
|  | | | *StAR* | | | | *TH* | | | *SLC25A4* | | | *MCCC1* | | *WT1* | | | *CDKL5* | | | *GNS* | | | | *MUT* | | | *ALDH7A1* | | | *GLYCTK* | | |
|  | | | *MMADHC* | | | | *SLC46A1* | | | *SLC19A1* | | | *SLC25A15* | | *MMADHC* | | |  | | |  | | | |  | | |  | | |  | | |
| Digestive system panel | | |  | | | |  | | |  | | |  | |  | | |  | | |  | | | |  | | |  | | |  | | |
|  | | | *NEUR0G3* | | | | *SPINT2* | | | *SLC26A3* | | | *EPCAM* | | *MY05B* | | | *CTLA4* | | | *MY09B* | | | | *BSND* | | | *APC* | | | *HLA-DQB1* | | |
|  | | | *ABCG8* | | | | *ABCB4* | | | *PRKCSH* | | | *SEC63* | | *CIRH1A* | | | *KRT8* | | | *KRT18* | | | | *IRGM* | | | *ADAM17* | | | *ATG16L1* | | |
|  | | | *ABCB1* | | | | *IRF5* | | | *IL23R* | | | *IL10RB* | | *IL10RA* | | | *NOD2* | | | *IL6* | | | | *PRSS1* | | | *SPINK 1* | | | *CFTR* | | |
|  | | | *RET* | | | | *ECE1* | | | *EDNRB* | | | *GDNF* | | *EDN3* | | | *NRTN* | | | *SMAD7* | | | | *IFNGR1* | | | *GIF* | | | *UGT1A1* | | |
|  | | | *TRMU* | | | | *FLNA* | | | *ABCC2* | | | *NCR3* | | *TYMP* | | | *TRIM37* | | | *SALL1* | | | | *SLC5A1* | | | *ACVRL1* | | | *SLC46A1* | | |
|  | | | *ALDOB* | | | | *MKKS* | | | *SAR1B* | | | *SCN9A* | | *ALDOB* | | |  | | |  | | | |  | | |  | | |  | | |
| Immune panel | | | | |  | | | |  | | | |  | |  | |  | | |  | | |  | |  | | |  | | |  | | |
|  | | | | | *ACP5* | | | | *CD19* | | | | *CYBA* | | *IL1RN* | | *NCF2* | | | *RNASEH2C* | | | *THBD* | | *ACTB* | | | *CD247* | | | *CYBB* | | |
|  | | | | | *IL21R* | | | | *NCF4* | | | | *RNF168* | | *TICAM1* | | *ADA* | | | *CD27* | | | *DCLRE1C* | | *IL2RA* | | | *NFKB2* | | | *RPSA* | | |
|  | | | | | *TINF2* | | | | *ADAR* | | | | *CD3D* | | *DKC1* | | *IL2RG* | | | *NFKBIA* | | | *RTEL1* | | *TLR3* | | | *AICDA* | | | *CD3E* | | |
|  | | | | | *DNMT3B* | | | | *IL36RN* | | | | *NHP2* | | *SAMHD1* | | *TMC6* | | | *AIRE* | | | *CD3G* | | *DOCK8* | | | *IL6* | | | *NLRP12* | | |
|  | | | | | *SBDS* | | | | *TMC8* | | | | *AK2* | | *CD40* | | *ELANE* | | | *IL7R* | | | *NLRP3* | | *SEMA3E* | | | *TNFRSF13B* | | | *AP3B1* | | |
|  | | | | | *CD40LG* | | | | *FADD* | | | | *IRAK4* | | *NOD2* | | *SERPING1* | | | *TNFRSF13C* | | | *APOL1* | | *CD46* | | | *FAS* | | | *IRF8* | | |
|  | | | | | *NOP10* | | | | *SH2D1A* | | | | *TNFRSF1A* | | *ATM* | | *CD59* | | | *FASLG* | | | *ISG15* | | *NRAS* | | | *SH3BP2* | | | *TNFRSF4* | | |
|  | | | | | *BLM* | | | | *CD79A* | | | | *FCN3* | | *ITCH* | | *ORAI1* | | | *SLC29A3* | | | *TNFSF12* | | *BLNK* | | | *CD79B* | | | *FERMT3* | | |
|  | | | | | *ITGB2* | | | | *PIK3CD* | | | | *SLC35C1* | | *TRAF3* | | *BTK* | | | *CD81* | | | *FOXN1* | | *ITK* | | | *PIK3R1* | | | *SLC37A4* | | |
|  | | | | | *TRAF3IP2* | | | | *C1QA* | | | | *CD8A* | | *FOXP3* | | *JAK3* | | | *PLCG2* | | | *SLC46A1* | | *TREX1* | | | *C1QB* | | | *CEBPE* | | |
|  | | | | | *FPR1* | | | | *KRAS* | | | | *PMS2* | | *SMARCAL1* | | *TTC7A* | | | *C1QC* | | | *CFB* | | *G6PC3* | | | *LAMTOR2* | | | *PNP* | | |
|  | | | | | *SP110* | | | | *TYK2* | | | | *C1R* | | *CFD* | | *GATA2* | | | *LCK* | | | *POLE* | | *SPINK5* | | | *UNC119* | | | *C1S* | | |
|  | | | | | *CFH* | | | | *GFI1* | | | | *LIG4* | | *PRF1* | | *STAT1* | | | *UNC13D* | | | *C2* | | *CFHR1* | | | *HAX1* | | | *LPIN2* | | |
|  | | | | | *PRKCD* | | | | *STAT2* | | | | *UNC93B1* | | *C3* | | *CFHR2* | | | *ICOS* | | | *LRBA* | | *PRKDC* | | | *STAT3* | | | *UNG* | | |
|  | | | | | *C4A* | | | | *CFHR3* | | | | *IFNG* | | *LYST* | | *PSMB8* | | | *STAT5B* | | | *USB1* | | *C4B* | | | *CFHR4* | | | *IFNGR1* | | |
| Name for TES Panels | | | | Genes involved in the panel | | | | | | | | | | | | | | | | | | | | | | | | | | | | | |
|  | | | | | *MAGT1* | | | | *PSTPIP1* | | | | *STIM1* | | *VPS13B* | | *C5* | | | *CFHR5* | | | *IFNGR2* | | *MALT1* | | | *PTPRC* | | | *STK4* | | |
|  | | | *VPS45* | | | | *C6* | | | *CFI* | | | *IGLL1* | | *MASP1* | | | *RAB27A* | | | *STX11* | | | | *WAS* | | | *C7* | | | *CFP* | | |
|  | | | *IKBKB* | | | | *MASP2* | | | *RAC2* | | | *STXBP2* | | *WIPF1* | | | *C8A* | | | *CHD7* | | | | *IKBKG* | | | *MCM4* | | | *RAG1* | | |
|  | | | *TAP1* | | | | *XIAP* | | | *C8B* | | | *CIITA* | | *IKZF1* | | | *MEFV* | | | *RAG2* | | | | *TAP2* | | | *ZAP70* | | | *C8G* | | |
|  | | | *CLEC7A* | | | | *IL10* | | | *MRE11A* | | | *RBCK1* | | *TAPBP* | | | *ZBTB24* | | | *C9* | | | | *COLEC11* | | | *IL10RA* | | | *MS4A1* | | |
|  | | | *RFX5* | | | | *TAZ* | | | *CARD11* | | | *CORO1A* | | *IL10RB* | | | *MTHFD1* | | | *RFXANK* | | | | *TBK1* | | | *CARD14* | | | *CR2* | | |
|  | | | *IL12B* | | | | *MVK* | | | *RFXAP* | | | *TBX1* | | *CARD9* | | | *CSF2RA* | | | *IL12RB1* | | | | *MYD88* | | | *RHOH* | | | *TCF3* | | |
|  | | | *CASP10* | | | | *CTSC* | | | *IL17F* | | | *NBN* | | *RNASEH2A* | | | *TCN2* | | | *CASP8* | | | | *CXCR4* | | | *IL17RA* | | | *NCF1* | | |
|  | | | *RNASEH2B* | | | | *TERT* | | |  | | |  | |  | | |  | | |  | | | |  | | |  | | |  | | |

Abbreviation: TES: Targeted Exome Sequencing.

**Definitions of immune-related conditions in HPO terms**

In this study, we selected 8 immune-related conditions. These phenotypes occur most frequently in NICUs. They was selected based on extensive literature research and the following criteria: i) they potentially discriminate between healthy subjects and patients; and ii) some specific biomarkers can be measured in the blood or other samples collected according to generally accepted and applicable procedures for bio sample collection. The phenotypes of the affected infants were further translated into Human Phenotype Ontology (HPO) terms. Many infants had more than one phenotype.

| HPO term | HPO ID | Clinical meaning |
| --- | --- | --- |
| atopic dermatitis    thrombocytopenia  autoimmune hemolytic anemia  Recurrent infections in infancy and early childhood  Failure to thrive secondary to recurrent infections  Recurrent infections  Intractable diarrhea  protracted diarrhea  Leukocytosis  Leukopenia  Abnormal immunoglobulin level  family history | HP:0001047  HP:0001873  HP:0001890  HP:0005437  HP:0008866  HP:0002719  HP:0002041  HP:0004385  HP:0001974  HP:0001882  HP:0010701  HP:0032316 | A chronic inflammatory genetically determined disease of the skin manifested by lichenification, excoriation, and crusting, mainly on the flexural surfaces of the elbow and knee.  A reduction in the number of circulating `thrombocytes`.  An autoimmune form of `hemolytic anemia`.  Recurrent infections at an early age with improvement in later childhood.  Insufficient weight gain or inappropriate weight loss for a child that is attributed to an endogenous recurrent infections.  Increased susceptibility to microbial infections, as manifested by recurrent episodes of infection.  Abnormally increased frequency of loose or watery bowel movements.  Severe, progressive infantile onset inflammatory bowel disease  An abnormal increase in the number of leukocytes in the blood.  An abnormal decreased number of leukocytes in the blood.  An abnormal deviation from normal levels of immunoglobulins in blood.  Information about close relatives of an individual who is the proband of a study or who is being investigated with the goal of identifying a medical diagnosis. Usually, the family history includes information from three generations of relatives, including children, brothers and sisters, parents, aunts and uncles, nieces and nephews, grandparents, and cousins. |

Note: This description, based on cases published in biomedical literatures and our cohort, uses the phenotypic abnormalities referenced in the Human Phenotype Ontology (HPO). Listed under each header are the unique HPO-based abnormalities that were reported among the cohort of 143 infants in our study. In addition, for the description for the term “increased susceptibility to life-threatening infections”, we refer to those patients if they have sepsis or sepsis shock, or if they need ventilation support or vasopressor therapy or present organ dysfunction, coupled with suspected infection.

Abbreviations: HPO, human phenotype ontology; HP, human phenotype.

**Predictors of a Positive Diagnosis of PID**

Odds Ratio (OR) was calculated to verify characteristics of patients in predicting a PID diagnosis.

**Independent Predictors of 180-day mortality**

To identify independent predictors of 180-day mortality, we started with a full model including variables (characteristics of patients, their PID or non-PID subgroup, and their pathway subgroup). A multivariable logistic regression analysis was performed to evaluate the independent significance.

Risk factors of 180-day mortality include: gender; the age of onset; gestational age; birth weight; prenatal anomalies; family history; age of testing; length of stay; turnaround time; PID diagnosis; JAK/STATopathy; immune-related phenotypes: life-threatening infections, intractable diarrhea, atopic dermatitis, failure to thrive, thrombocytopenia, leukocytosis/neutropenia, recurrent infections.

**The Diagnostic Performance of the Independent Predictors in Identifying the Patients with a180-day mortality in NICU**

We analyzed receiver operating characteristic (ROC) curves according to the values of sensitivity, specificity, and Youden’s index. We defined areas under the ROC curves (AUCs) < 0.7 as having poor discriminatory value, 0.7-0.8 as minimally accurate, 0.8-0.9 as having good accuracy, and > 0.9 as having excellent accuracy. For those variables with a Youden’s index >0.7, we determined their optimal cutoff value that differentiated patients with a 180-day mortality and those without.

Supplementary table 1 Profiles of 72 patients molecularly diagnosed in three NICUs.

| ID | Gender | PID subgroup | JAK-STAT subgroup | Theage of onset | Immune-related conditions | Family members tests | Gene | cDNA mutation | Amino acid alteration | Molecular Diagnosis | Inheritance pattern | Zygosity | Parent of Origin | Consanguinity | Follow-up |
| --- | --- | --- | --- | --- | --- | --- | --- | --- | --- | --- | --- | --- | --- | --- | --- |
| 1 | M | PID group | JS group | 60 | life-threatening infections;atopic dermatitis; thrombocytopenia; anemia | Trio | *JAK3* (NM_000215.3) | c.3050T>C;  c.1744C>T | p.Leu1017Pro;  p.Arg582Trp | JAK3 deficiency  [MIM: 600173] | AR | het | Inherited (from father + mother) | N | death at age of 111 day after birth |
| 2 | F | non-PID group | JS group | 1 | recurrent infections | Trio | *HRAS* (NM_005343.4) | c.37G>T | p. Gly13Cys | Costello syndrome [MIM:16835863] | AD | het | De novo | N | alive |
| 3 | M | non-PID group | JS group | 2 | thrombocytopenia | Trio | *CREBBP* (NM_004380.3) | c.4990C>T | p.Arg1664Cys | Rubinstein-Taybi syndrome 1 [MIM:180849] | AD | het | De novo | N | alive |
| 4 | M | non-PID group | JS group | 32 | recurrent infections | Trio | *RAF1* (NM_002880.3) | c.770C>T | p.Ser257Leu | Noonan syndrome 5 [MIM:611553] | AD | het | De novo | N | alive |
| 5 | M | non-PID group | JS group | 5 | recurrent infections; protracted diarrhea; thrombocytopenia | Trio | *PTPN11* (NM_002834.3) | c.1517A>C | p.Gln506Pro | Noonan syndrome 1 [MIM:163950];  LEOPARD syndrome 1 [MIM:151100]; | AD | het | De novo | N | death at age of 146 day after birth |
| 6 | M | non-PID group | NJS group | 0 | recurrent infections; atopic dermatitis; | Trio | *EDA* (NM_001399.5) | c.730C>T | p.Arg244* | Ectodermal dysplasia 1, hypohidrotic, X-linked [MIM:305100] | XR | hemi | Inherited  (from mother) | N | alive |
| 7 | F | non-PID group | NJS group | 0 | atopic dermatitis | Proband | *COL17A1* (NM_000494.3) | c.2363dupG;  c.3301C>T | p.Leu789Thrfs*13  p.Arg1101Cys | Epidermolysis bullosa, junctional, localisata variant [MIM:226650] | AR | het | Unknown  (only proband) | N | alive |
| 8 | M | non-PID group | NJS group | 0 | atopic dermatitis | Trio | *COL7A1* (NM_000094.3) | c.3625_3635del;3625_3635del | p.? | Epidermolysis bullosa dystrophica, [MIM:226600] | AR | hom | Inherited (from father + mother) | N | alive |
| 9 | F | non-PID group | NJS group | 0 | atopic dermatitis | Trio | *COL7A1* (NM_000094.3) | c.6181-2A>G | p.? | Epidermolysis bullosa dystrophica, AD [MIM:131750] | AD | het | Inherited  (from father) | N | alive |
| 10 | M | non-PID group | NJS group | 0 | life-threatening infections; atopic dermatitis | Trio | *COL7A1* (NM_000094.3) | c.5980-2A>G | p.? | Epidermolysis bullosa dystrophica, AD [MIM:131750] | AD | het | De novo | N | alive |
| 11 | F | non-PID group | NJS group | 0 | atopic dermatitis | Trio | *COL7A1* (NM_000094.3) | c.6929G>A | p. Gly2310Glu | Epidermolysis bullosa dystrophica, AD [MIM:131750] | AD | het | Inherited  (from mother) | N | alive |

Supplementary table 1 Continued

| ID | Gender | PID subgroup | JAK-STAT subgroup | The  age of onset | Immune-related conditions | Family members tests | Gene | cDNA mutation | Amino acid alteration | Molecular Diagnosis | Inheritance pattern | Zygosity | Parent of Origin | Consanguinity | Follow-up |
| --- | --- | --- | --- | --- | --- | --- | --- | --- | --- | --- | --- | --- | --- | --- | --- |
| 12 | F | PID group | JS group | 6 | life-threatening infections; protracted diarrhea; thrombocytopenia; anemia | Trio | *IL10RA* (NM_001558.4) | c.301C>T;  c.1283delC | p.Arg101Trp;  p.Pro428Argfs*20 | IL-10Ra deficiency  [MIM: 146933] | AR | het | Inherited (from father + mother) | N | death at age of 26 day after birth |
| 13 | M | PID group | JS group | 12 | recurrent infections; protracted diarrhea; atopic dermatitis; failure to thrive secondary to recurrent infections or protracted diarrhea | Trio | *IL10RA* (NM_001558.4) | c.301C>T;  c.537G>A | p.Arg101Trp;  p.= | IL-10Ra deficiency  [MIM: 146933] | AR | het | Inherited (from father + mother) | N | alive |
| 14 | M | PID group | JS group | 8 | life-threatening infections; protracted diarrhea | Trio | *IL10RA* (NM_001558.4) | c.106G>A;  c.299T>G | p.Ala36Thr;  p.Val100Gly | IL-10Ra deficiency  [MIM: 146933] | AR | het | Inherited (from father + mother) | N | alive |
| 15 | M | PID group | JS group | 90 | life-threatening infections; recurrent infections; thrombocytopenia; anemia | Trio | *IL2RG* (NM_000206.3) | c.421C>T | p.Gln141* | γc deficiency (common  gamma chain SCID,  CD132 deficiency)  [MIM: 308380] | XR | hemi | Inherited  (from mother) | N | death at age of 140 day after birth |
| 16 | M | PID group | JS group | 0 | recurrent infections; failure to thrive secondary to recurrent infections; thrombocytopenia | Trio | *CXCR4* (NM_003467.2) | c.685T>A | p.Ser229Thr | WHIM (warts,  hypogammaglobulinemia,  infections, myelokathexis)  syndrom [MIM: 162643] | AD | het | Inherited  (from mother) | N | alive |
| 17 | M | PID group | NJS group | 7 | life-threatening infections; recurrent infections; atopic dermatitis; failure to thrive secondary to recurrent infections; anemia | Trio | *ITGB2* (NM_000211.5) | c.817G>A; | p.Gly273Arg | Leukocyte adhesion  deficiency type 1 (LAD1)  [MIM: 600065] | AR | hom | De novo / Inherited  (from father) | N | alive |
| 18 | F | non-PID group | NJS group | 19 | life-threatening infections; anemia | Trio | *ABCC8* (NM_000352.6) | c.1671+2T>C | p.? | Familial hyperinsulinemic hypoglycemia [MIM:256450] | AD | het | Inherited  (from mother) | N | alive |
| 19 | F | non-PID group | NJS group | 0 | life-threatening infections; atopic dermatitis | Trio+1sister | *TGM1* (NM_000359.3) | c.1130G>A;  c.871G>A | p.Cys377Tyr ;  p.Gly291Ser | Ichthyosis [MIM:242300] | AR | het | Inherited (from father + mother) | N | alive |

Supplementary table 1 Continued

| ID | Gender | PID subgroup | JAK-STAT subgroup | The  age of onset | Immune-related conditions | Family members tests | Gene | cDNA mutation | Amino acid alteration | Molecular Diagnosis | Inheritance pattern | Zygosity | Parent of Origin | Consanguinity | Follow-up |
| --- | --- | --- | --- | --- | --- | --- | --- | --- | --- | --- | --- | --- | --- | --- | --- |
| 20 | M | non-PID group | NJS group | 4 | life-threatening infections; thrombocytopenia | Trio | *FLI1* (NM_002017.5) | FLI1 heterozygous del | (-) | Bleeding disorder, platelet-type, 21 [MIM:617443] | AD | het | De novo | N | death at age of 14 day after birth |
| 21 | M | non-PID group | NJS group | 0 | life-threatening infections; recurrent infections; protracted diarrhea; failure to thrive secondary to recurrent infections or protracted diarrhea; thrombocytopenia; anemia | Trio | *EPHB4* (NM_004444.5) | c.2354G>A | p.Arg785Gln | Lymphatic malformation 7 [MIM:617300] | AD | het | De novo | N | alive |
| 22 | M | non-PID group | NJS group | 97 | recurrent infections; anemia | Trio | *ALPL* (NM_000478.6) | c.18del;  c.1101_1103del | p.Val7Tyrfs*12;  p.Ser368del | Hypophosphatasia, infantile forms [MIM:241500] | AR | het | Inherited (from father + mother) | N | death at age of 176 day after birth |
| 23 | M | non-PID group | NJS group | 71 | life-threatening infections; recurrent infections; failure to thrive secondary to recurrent infections; anemia | Proband | 16p11.2p12.2 del | chr16p11.2p12.2 del | (-) | Chromosome 16p12.2-p11.2 deletion syndrome [MIM:613604] | Isolated cases | het | Unknown  (only proband) | N | death at age of 178 day after birth |
| 24 | M | non-PID group | NJS group | 68 | neutropenia; thrombocytopenia; anemia | Trio | *MMACHC* (NM_015506.3) | c.217C>T;  c.609G>A | p.Arg73*;  p.Trp203* | Methylmalonic aciduria and homocystinuria, cblC type [MIM:277400] | AR | het | Inherited (from father + mother) | N | death at age of 76 day after birth |
| 25 | F | non-PID group | NJS group | 5 | life-threatening infections; thrombocytopenia; anemia | Trio+1sister | *ABCC8* (NM_000352.3) | c.4412-13G>A;  c.2992C>T | p.?  p.Arg998* | Hypoglycaemia, persistent hyperinsulinaemic [MIM:256450] | AR | het | Inherited (from father + mother) | N | death at age of 37 day after birth |
| 26 | F | non-PID group | NJS group | 37 | life-threatening infections; protracted diarrhea; anemia | Trio | *CPS1* (NM_001875.4) | c.2162G>A;  c.2938G>A | p.Arg721Gln;  p.Gly980Ser | Carbamoylphosphate synthetase I deficiency [MIM:237300] | AR | het | Inherited (from father + mother) | N | alive |
| 27 | M | non-PID group | NJS group | 0 | life-threatening infections; anemia | Proband | *HBG2* (NM_000184.3) | c.190C> T | p.His64Tyr | Fetal hemoglobin quantitative trait locus1 [MIM:141749] | AD | het | Unknown  (only proband) | N | alive |
| 28 | M | PID group | NJS group | 0 | life-threatening infections | Proband | 22q11.2del | chr22q11.2del | (-) | Chromosome 22q11.2  deletion syndrome  [MIM: 602054] | AD | het | Unknown  (only proband) | N | alive |

Supplementary table 1 Continued

| ID | Gender | PID subgroup | JAK-STAT subgroup | The  age of onset | Immune-related conditions | Family members tests | Gene | cDNA mutation | Amino acid alteration | Molecular Diagnosis | Inheritance pattern | Zygosity | Parent of Origin | Consanguinity | Follow-up |
| --- | --- | --- | --- | --- | --- | --- | --- | --- | --- | --- | --- | --- | --- | --- | --- |
| 29 | M | non-PID group | NJS group | 17 | recurrent infections; failure to thrive secondary to recurrent infections; thrombocytopenia; anemia | Trio | *MMACHC* (NM_015506.3) | c.658_660del | p.Lys220del | Methylmalonic aciduria and homocystinuria, cblC type [MIM:277400] | AR | hom | Inherited (from father + mother) | N | alive |
| 30 | M | non-PID group | NJS group | 64 | recurrent infections; anemia | Trio | *OTC* (NM_000531.6) | c.540G>C | p.Gln180His | Ornithine transcarbamylase deficiency [MIM:311250] | XR | het | Inherited  (from mother) | N | death at age of 177 day after birth |
| 31 | M | non-PID group | NJS group | 20 | protracted diarrhea | Trio | *PCSK1* (NM_000439.5) | c.1777G>A; | p.Gly593Arg | Obesity with impaired prohormone processing [MIM:600955] | AR | hom | Inherited (from father + mother) | N | alive |
| 32 | M | non-PID group | NJS group | 0 | recurrent infections; atopic dermatitis | Proband+father | *ALOX12B* (NM_001139.3) | c.1405C>T;  c.163_173del | p.Arg469Trp  p.Val55Serfs*5 | Ichthyosis, congenital, autosomal recessive 2[MIM:242100] | AR | het | Inherited  (from father) | N | alive |
| 33 | M | non-PID group | NJS group | 17 | life-threatening infections; protracted diarrhea; failure to thrive secondary to protracted diarrhea; thrombocytopenia; anemia | Trio | *EPCAM* (NM_002354.3) | c.96C>A;  c.823delG | p.Tyr32*;  p.Val275Trpfs*2 | Diarrhea 5, with tufting enteropathy, congenital [MIM:613217] | AR | het | Inherited (from father + mother) | N | alive |
| 34 | M | non-PID group | NJS group | 74 | anemia | Trio | *AQP2* (NM_000486.5) | c.454C> T;  c.202A>T | p.Arg152Cys ;  p.Asn68Tyr | Diabetes insipidus, nephrogenic [MIM:125800] | AR | het | Inherited (from father + mother) | N | alive |
| 35 | M | non-PID group | NJS group | 13 | anemia | Proband | *F13A1* (NM_000129.4) | c.2015G>A;  c.1352_1353del | p.Gly672Glu;  p.His451Argfs*29 | Factor XIIIA deficiency [MIM:613225] | AR | het | Unknown  (only proband) | N | alive |
| 36 | F | non-PID group | NJS group | 62 | life-threatening infections; atopic dermatitis | Trio | *TNFRSF13B* (NM_012452.3) | c.306del | p.Phe103Serfs*10 | Immunodeficiency, common variable, 2  [MIM:240500] | AD, AR | het | Inherited  (from mother) | N | alive |
| 37 | F | non-PID group | NJS group | 24 | neutropenia; thrombocytopenia ;anemia | Trio | 11q24.1-q25 del | chr11q24.1q25del | (-) | Jacobsen syndrome [MIM:147791] | Isolated cases | het | De novo | N | alive |
| 38 | F | non-PID group | NJS group | 90 | recurrent infections | Proband | *STAR* (NM_000349.3) | c.772C>T;  c.556A>G | p.Gln258*;  p.Ser186Gly | Lipoid adrenal hyperplasia [MIM:201710] | AR | het | Unknown  (only proband) | N | alive |

Supplementary table 1 Continued

| ID | Gender | PID subgroup | JAK-STAT subgroup | The  age of onset | Immune-related conditions | Family members tests | Gene | cDNA mutation | Amino acid alteration | Molecular Diagnosis | Inheritance pattern | Zygosity | Parent of Origin | Consanguinity | Follow-up |
| --- | --- | --- | --- | --- | --- | --- | --- | --- | --- | --- | --- | --- | --- | --- | --- |
| 39 | M | PID group | NJS group | 39 | life-threatening infections; recurrent infections; failure to thrive secondary to recurrent infections; thrombocytopenia | Trio | *IKBKG*  (NM_003639.4) | c.1110delinsTT | p.Ala371Cysfs*24 | EDA-ID due to IKBKG  deficiency  [MIM: 300248] | XR | hemi | Inherited  (from mother) | N | death at age of 60 day after birth |
| 40 | M | non-PID group | NJS group | 5 | life-threatening infections | Trio | *G6PD* (NM_000402.3) | c.185A>G | p.His62Arg | Hemolytic anemia, G6PD deficient (favism)[MIM:300908] | XD | hemi | Inherited  (from mother) | N | alive |
| 41 | F | PID group | NJS group | 3 | atopic dermatitis | Trio | *IKBKG* (NM_003639.4) | Exon 4-10 del | (-) | EDA-ID due to IKBKG  deficiency  [MIM: 300248] | XD | het | Inherited  (from mother) | N | alive |
| 42 | M | non-PID group | NJS group | 5 | recurrent infections | Trio | *CHRNA2* (NM_000742.4) | c.1073G>T | p.Ser358Ile | Epilepsy, nocturnal frontal lobe, 4 [MIM:610353] | AD | het | Inherited  (from mother) | N | alive |
| 43 | M | non-PID group | NJS group | 0 | recurrent infections; anemia | Trio | 15q11.2q13.1 del | chr15q11.2q13.1del | (-) | Prader-Willi syndrome due to paternal deletion of 15q11q13 type 2 [MIM:176270] | Imprinted disease | het | Inherited  (from father) | N | alive |
| 44 | M | non-PID group | NJS group | 0 | thrombocytopenia | Proband | *IGHMBP2* (NM_002180.3) | c.2356del;  c.344C>T | p.Ala786Profs*45;  p.Thr115Met | Neuronopathy, distal hereditary motor, type VI [MIM:604320]; Charcot-Marie-Tooth disease, axonal, type 2S[MIM:616155] | AR | het | Unknown  (only proband) | N | alive |
| 45 | F | non-PID group | NJS group | 0 | life-threatening infections; thrombocytopenia; anemia | Trio | *MYH7* (NM_000257.4) | c.5655G>A | p.= | Cardiomyopathy, dilated, 1S,Left ventricular noncompaction 5 [MIM:613426]; Cardiomyopathy, hypertrophic, 1 [MIM:192600];  Laing distal myopathy [MIM:160500]; Myopathy, myosin storage, autosomal dominant[MIM:608358] | AD | het | Inherited  (from father) | N | death at age of 7 day after birth |

| ID | Gender | PID subgroup | JAK-STAT subgroup | The  age of onset | Immune-related conditions | Family members tests | Gene | cDNA mutation | Amino acid alteration | Molecular Diagnosis | Inheritance pattern | Zygosity | Parent of Origin | Consanguinity | Follow-up |
| --- | --- | --- | --- | --- | --- | --- | --- | --- | --- | --- | --- | --- | --- | --- | --- |
| 46 | F | PID group | NJS group | 10 | life-threatening infections; atopic dermatitis; anemia | Trio | *SPINK5* (NM_006846.4) | c.377_378del;  c.2468dup | p.Tyr126*;  p.Lys824Glufs*4 | Comel-Netherton  syndrome [MIM: 605010] | AR | het | Inherited (from father + mother) | N | death at age of 40 day after birth |
| 47 | F | PID group | NJS group | 1 | atopic dermatitis | Trio | *IKBKG* (NM_003639.4) | Exon 4-10 del | (-) | EDA-ID due to IKBKG  deficiency  [MIM: 300248] | XD | het | Inherited  (from mother) | N | alive |
| 48 | M | PID group | NJS group | 1 | recurrent infections; anemia | Trio | *CHD7* (NM_017780.4) | c.6292C>T | p.Arg2098* | CHARGE syndrome  due to CHD7  deficiency [MIM: 608892] | AD | het | De novo | N | alive |
| 49 | M | non-PID group | NJS group | 26 | atopic dermatitis; anemia | Trio | *AMER1* (NM_152424.4) | c.2082C>A | p.Asp694Glu | Osteopathia striata with cranial sclerosis [MIM:300373] | XD | het | Inherited  (from mother) | N | death at age of 105 day after birth |
| 50 | F | non-PID group | NJS group | 2 | thrombocytopenia | Trio | *RPS19* (NM_001022.3) | c.3G>A | p.? | Diamond-Blackfan anemia 1 [MIM:105650] | AD | het | De novo | N | alive |
| 51 | F | non-PID group | NJS group | 0 | life-threatening infections | Proband | Chromosome 18 duplication | chromosome 18 duplication | (-) | Trisomy 18 syndrome [MIM:601161] | Isolated cases | het | Unknown  (only proband) | N | alive |
| 52 | M | PID group | NJS group | 28 | life-threatening infections; protracted diarrhea | Trio | *KMT2D* (NM_003482.3) | c.14382G>A | p.= | Kabuki syndrome 1 due  to KMT2D deficiency  [MIM: 602113] | AD | het | De novo | N | alive |
| 53 | F | non-PID group | NJS group | 19 | recurrent infections | Proband | *EFTUD2* (NM_004247.3) | c.869+1G>A | p.? | Mandibulofacial dysostosis, Guion-Almeida type [MIM:610536] | AD | het | Unknown  (only proband) | N | alive |
| 54 | M | non-PID group | NJS group | 0 | life-threatening infections; thrombocytopenia | Proband | Distal duplication 16q | Distal duplication 16q | (-) | Distal trisomy 16q | Isolated cases | het | Unknown  (only proband) | N | death at age of 15 day after birth |
| 55 | M | non-PID group | NJS group | 14 | life-threatening infections; recurrent infections; thrombocytopenia | Trio | *KCNJ11* (NM_000525.3) | c.1006del | p.Thr336Profs*24 | Hyperinsulinemic hypoglycemia, familial, 2 [MIM:601820] | AR | hom | Inherited (from father + mother) | N | alive |
| 56 | M | non-PID group | NJS group | 21 | protracted diarrhea; failure to thrive secondary to protracted diarrhea | Proband | *MYO5B* (NM_001080467.2) | c.2T>C;  c.1201C>G | p.?;  p.Arg401Gly | Microvillus inclusion disease [MIM:251850] | AR | het | Unknown  (only proband) | N | death at age of 30 day after birth |

Supplementary table 1 Continued

Supplementary table 1 Continued

| ID | Gender | PID subgroup | JAK-STAT subgroup | The  age of onset | Immune-related conditions | Family members tests | Gene | cDNA mutation | Amino acid alteration | Molecular Diagnosis | Inheritance pattern | Zygosity | Parent of Origin | Consanguinity | Follow-up |
| --- | --- | --- | --- | --- | --- | --- | --- | --- | --- | --- | --- | --- | --- | --- | --- |
| 57 | F | non-PID group | NJS group | 2 | thrombocytopenia | Trio | *GP1BA* (NM_000173.6) | c.737G>T | p.Trp246Leu | Bernard-Soulier syndrome, type A2 (dominant) [MIM:153670] | AD | het | Inherited  (from father) | N | alive |
| 58 | F | non-PID group | NJS group | 19 | life-threatening infections; recurrent infections; failure to thrive secondary to recurrent infections | Proband | 21q22.12q22.13 del | chr21q22.12q22.13del | (-) | - | Isolated cases | het | Unknown  (only proband) | N | alive |
| 59 | F | non-PID group | NJS group | 7 | thrombocytopenia | Proband | 11q24.1q25del | chr11q24.1q25del | (-) | Jacobsen syndrome [MIM:147791] | Isolated cases | het | Unknown  (only proband) | N | alive |
| 60 | M | non-PID group | NJS group | 8 | atopic dermatitis | Proband | Xp11.23p11.22dup | chrXp11.23p11.22dup | (-) | Chromosome Xp11.23-p11.22 duplication syndrome  [MIM:300801] | XD | het | Unknown  (only proband) | N | alive |
| 61 | M | PID group | JS group | 65 | life-threatening infections; recurrent infections; protracted diarrhea; failure to thrive secondary to recurrent infections or protracted diarrhea; thrombocytopenia; anemia | Trio | *IL10RA* (NM_001558.4) | c.251C>T; c.537G>A | p.Thr84Ile;  p.= | IL-10Ra deficiency  [MIM: 146933] | AR | het | Inherited  (from mother) | N | alive |
| 62 | M | non-PID group | JS group | 3 | life-threatening infections | Trio | *PTPN11* (NM_002834.3) | c.487G>A | p.Gly163Ser | LEOPARD syndrome [MIM:151100] | AD | het | Inherited (from father + mother) | N | alive |
| 63 | M | PID group | NJS group | 61 | life-threatening infections; neutropenia; thrombocytopenia | Trio | *UNC13D* (NM_199242.3) | c.766C>T;  c.640C>T | p.Arg256*;  p.Arg214* | UNC13D/Munc13-4  deficiency (FHL3)  [MIM: 608897] | AR | het | Inherited  (from mother) | N | death at age of 169 day after birth |
| 64 | M | PID group | NJS group | 46 | life-threatening infections; recurrent infections; atopic dermatitis; anemia | Trio | *IKBKG* (NM_001099856.6) | c.662T>C | p.Leu221Pro | EDA-ID due to IKBKG  deficiency  [MIM: 300248] | XR | hemi | Inherited  (from mother) | N | death at age of 137 day after birth |
| 65 | M | PID group | NJS group | 28 | atopic dermatitis | Proband | *IKBKG* (NM_003639.4) | Exon 4-10 del | (-) | EDA-ID due to IKBKG  deficiency  [MIM: 300248] | XD | hemi | Unknown  (only proband) | N | alive |

Supplementary table 1 Continued

| ID | Gender | PID subgroup | JAK-STAT subgroup | The  age of onset | Immune-related conditions | Family members tests | Gene | cDNA mutation | Amino acid alteration | Molecular Diagnosis | Inheritance pattern | Zygosity | Parent of Origin | Consanguinity | Follow-up |
| --- | --- | --- | --- | --- | --- | --- | --- | --- | --- | --- | --- | --- | --- | --- | --- |
| 66 | F | PID group | NJS group | 73 | recurrent infections; anemia | Trio | *KMT2D* (NM_003482.3) | c.11017del | p.Asn3674Ilefs*75 | Kabuki syndrome 1 due  to KMT2D deficiency  [MIM: 602113] | AD | het | De novo | N | alive |
| 67 | F | PID group | NJS group | 0 | atopic dermatitis | Trio | *IKBKG* (NM_003639.4) | Exon 4-10 del | (-) | EDA-ID due to IKBKG  deficiency  [MIM: 300248] | XD | het | Inherited  (from mother) | N | alive |
| 68 | M | PID group | NJS group | 89 | life-threatening infections; anemia | Trio | *POLE* (NM_006231.4) | c.1181_1182del;  c.3587C>T | p.Gln394Argfs*29;  p.Thr1196Met | POLE1 (polymerase ε  subunit 1) deficiency  (FILS syndrome)  [MIM: 174762] | AR | het | Inherited (from father + mother) | N | alive |
| 69 | M | PID group | NJS group | 19 | life-threatening infections; atopic dermatitis | Trio | *CYBB*  (NM_000397.4) | c.1151+1G>A | p.? | X-linked chronic  granulomatous disease  (CGD), gp91phox  [MIM: 300481] | XR | het | Inherited  (from mother) | N | death at age of 167 day after birth |
| 70 | F | PID group | NJS group | 3 | life-threatening infections | Trio | *KMT2D* (NM_003482.3) | c.10595T>C | p.Ile3532Thr | Kabuki syndrome 1 due  to KMT2D deficiency  [MIM: 602113] | AD | het | De novo | N | alive |
| 71 | M | PID group | NJS group | 0 | life-threatening infections | Trio | *KMT2D* (NM_003482.3) | c.1967del | p.Leu656Argfs*274 | Kabuki syndrome 1 due  to KMT2D deficiency  [MIM: 602113] | AD | het | De novo | N | alive |
| 72 | F | PID group | NJS group | 0 | protracted diarrhea | Trio | *CHD7* (NM_017780.4) | c.6157C>T | p.Arg2053* | CHARGE syndrome  due to CHD7  deficiency  [MIM: 608892] | AD | het | De novo | N | alive |

Abbreviations: M, male; F, female; AR, autosomal recessive inheritance disease; XR, X-linked recessive inheritance disease; Het, heterozygous; Hemi, hemizygous; hom, homozygous; N, negative; PID: primary immunodeficiency; JS group, JAK-STAT subgroup; NJS group, non-JAK-STAT subgroup.

Supplementary table 2 Crude odds ratios of clinical phenotypes for identification of individuals with PIDs.

| immune-related phenotypes | Crude OR  (95% CI) | *P* Value |
| --- | --- | --- |
| life-threatening infections | 2.864 (1.047, 7.836) | **0.049** |
| intractable diarrhea | 2.158 (0.615, 7.574) | 0.320 |
| atopic dermatitis | 2.182 (0.766, 6.216) | 0.177 |
| failure to thrive | 1.708 (0.465 , 6.279) | 0.497 |
| leukocytosis/neutropenia | 0.938 (0.081, 10.875) | 0.725 |
| thrombocytopenia | 0.753 (0.261, 2.177) | 0.791 |
| autoimmune hemolytic anemia  recurrent infections | 1.387 (0.516, 3.727)  1.090 (0.395 ,3.008) | 0.614  1.000 |

Abbreviations: failure to thrive: failure to thrive secondary to recurrent infections or intractable diarrhea; PID: primary immunodeficiency; CI=confidence interval;

OR, odds ratio; significance considered when *P* < 0.05.

Supplementary table 3 Characteristics of routine hematology analysis and immunologic test results in 12 patients with JAK/STATopathy.

| ID  Number | PID subgroup | First  WBC | WBC  _Max | WBC  _Min | First  CRP | CRP  _Max | CRP_Min | First  Neu | Neu_Max | Neu_Min | First  HB | HB_Max | HB_Min | First PLT | PLT_Max | PLT_Min | CD3^+^  (%) | CD4^+^  (%) | CD8^+^  (%) | CD19^+^  (%) | CD16+(%) | IgA  g/L | IgM  g/L | IgG g/L | IgE IU/mL |
| --- | --- | --- | --- | --- | --- | --- | --- | --- | --- | --- | --- | --- | --- | --- | --- | --- | --- | --- | --- | --- | --- | --- | --- | --- | --- |
| 1 | PID | 15.2 | 21.29 | 1.9 | 5 | 109 | 5 | 11.8 | 17.91 | 0.52 | 119 | 119 | 70 | 231 | 231 | 19 | 4.37 | 3.81 | 0.18 | 71.48 | 8.18 | <0.25 | 0.28 | 2.76 | 9.84 |
| 2 | non-PID | 7.47 | 13.45 | - | 5 | 5 | - | 2.42 | 3.92 | 2.42 | 143 | - | 139 | 449 | 484 | 449 | - | - | - | - | - | - | - | - | - |
| 3 | non-PID | 5.73 | 5.73 | - | - | - | - | 2.88 |  | 2.88 | 126 | - | - | 35 |  | 35 | - | - | - | - | - | <0.0647 | <0.0438 | 16.3 | <4.73 |
| 4 | non-PID | 12.38 | 12.38 | - | - | - | - | 5.47 |  | 5.47 | 153 | - | - | 322 |  | 322 | - | - | - | - | - | - | - | - | - |
| 5 | non-PID | 15.73 | 31.59 | 10.42 | 1 | 8 | 1 | 6.86 | 12.83 | 3.65 | 128 | 133 | 95 | 30 | 177 | 24 | - | - | - | - | - | - | - | - | - |
| 12 | PID | 23.97 | 49.89 | 13.09 | 14 | 122 |  | 11.35 | 38.04 | 3.74 | 139 | 177 | 79 | 462 | 499 | 29 | 77.32 | 63.13 | 13.53 | 15.23 | 5.96 | 0.31 | 0.74 | 6.33 | 11.5 |
| 13 | PID | 14.6 | 25.31 | 8.87 | 21 | 93 | 5 | 6.6 | 13.15 | 3.16 | 99 | 120 | 76 | 458 | 512 | 317 | 82.75 | 50.03 | 30.91 | 12.95 | 2.8 | 0.29 | 0.26 | 8.97 | 5.84 |
| 14 | PID | 9.5 | 26.67 |  | 17 | 152 | 8 | 3.2 | 12.88 | 3.2 | 154 | 182 | 90 | 378 | 650 | 378 | 80.81 | 54 | 25.01 | 8.21 | 6.73 | 0.34 | 0.81 | 7.89 | 50.6 |
| 15 | PID | 15.35 | 15.35 | 4.13 | 9 | 160 |  | 11.66 |  | 3.3 | 95 | 98 | 63 | 310 | - | 49 | 0.51 | 0.34 | 0.28 | 94.11 | 2.61 | <0.26 | <0.17 | 0.83 | <4.66 |
| 16 | PID | 16.4 | 16.4 | 4.2 | 33 |  | 6 | 2.25 |  | 0.55 | 64 | 104 | - | 42 | - | 15 | - | - | - | - | - | 0.71 | 0.82 | 10.1 | 88.5 |
| 61 | PID | 14.8 | 29.16 | 5.1 | 141 | 160 | 5 | 6.1 | 18.14 | 2.5 | 75 | 148 | 73 | 575 | - | 29 | 54.41 | 46.02 | 7.93 | 28.74 | 14.68 | 1.09 | 0.5 | 13.3 | 73.9 |
| 62 | non-PID | 10.91 | 18.03 | - | 5 | - | - | 6.43 | 8.62 | 6.43 | 145 | 157 | - | 263 | 460 | 263 | - | - | - | - | - | - | - | - | - |

Abbreviations: PID, primary immunodeficiency.

Note: references for routine hematology analysis and immunologic tests. WBC, 4-10 10^9^/L; CRP, <8 mg/L; Neu, 1.4-6.5 10^9^/L; HB, 110-160 g/L; PLT 100-300 10^9^/L; CD3^+^%, 60.8-75.4%;

CD4^+^%, 29.4-45.8%; CD8^+^%, 18.2-32.8%; CD19^+^%, 6.8-15.8%; CD16^+^%, 9.5-23.5%; IgA, 0.7-4.0g/L; IgM, 0.4-2.3g/L; IgG, 7-16.0g/L; IgE, 0-100 IU/m.

Supplementary table 4 Multiple Logistic Analysis of Risk Factors of 180-day mortality.

| Risk Factors | b | SE (b) | Wald x2 | *P* Value | OR | 95% CI |
| --- | --- | --- | --- | --- | --- | --- |
| The age of onset | -0.025 | 0.010 | 6.610 | **0.010** | 0.976 | 0.957，0.994 |
| PID diagnosis | 0.208 | 0.606 | 0.118 | 0.731 | 1.232 | 0.376，4.038 |
| JAK/STATopathy | -0.301 | 0.753 | 0.160 | 0.690 | 0.740 | 0.169，3.239 |

Abbreviations: PID, primary immunodeficiency; CI, confidence interval; OR, odds ratio, SE, standard error.


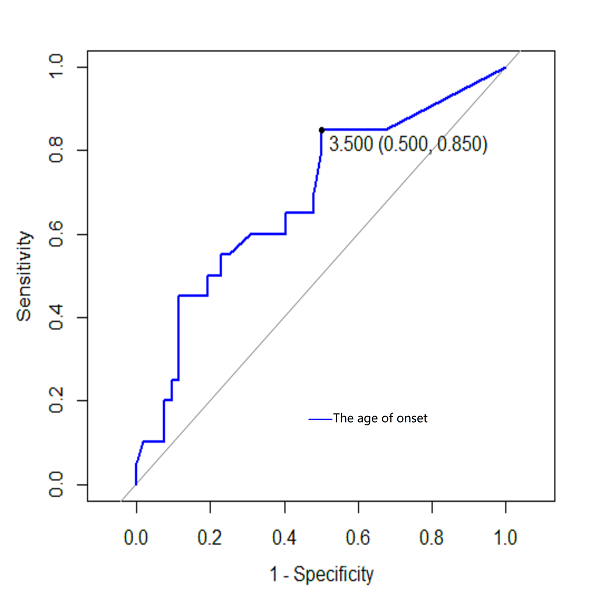


Supplementary figure 1 The age of onset of diseases with a genetic etiology for 180-day mortality by receiver operating characteristic (ROC) curves. The area under the curve (AUC) for the age of onset was 0.7001. Cutoff point: 3.5 days of age.


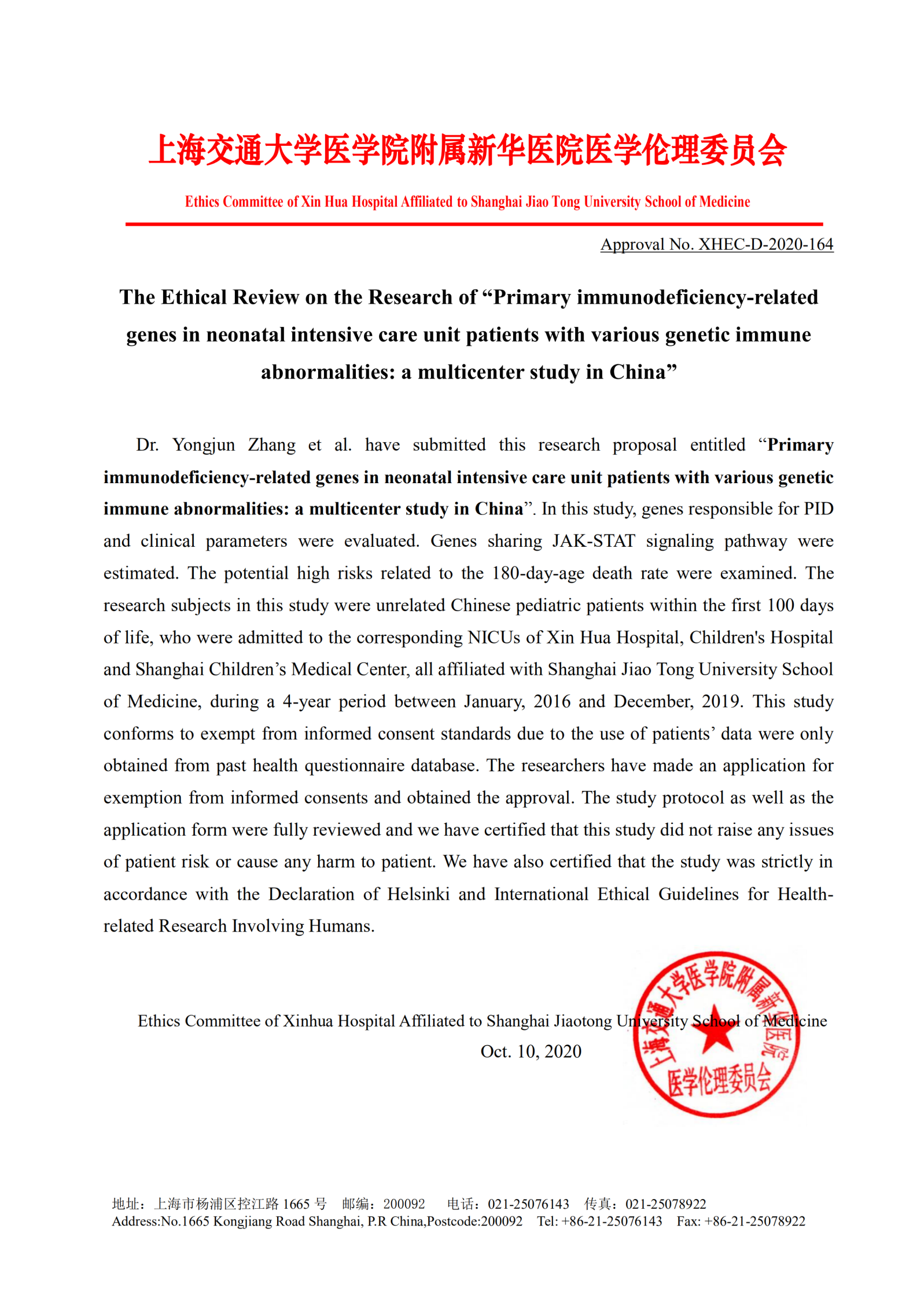

Supplement: Supplementary file 1 [file CTI2-10-e1266-s001.docx]
